# Latent Cytomegalovirus-Driven Recruitment of Activated CD4+ T Cells Promotes Virus Reactivation

**DOI:** 10.3389/fimmu.2021.657945

**Published:** 2021-04-12

**Authors:** Sarah E. Jackson, Kevin C. Chen, Ian J. Groves, George X. Sedikides, Amar Gandhi, Charlotte J. Houldcroft, Emma L. Poole, Inmaculada Montanuy, Gavin M. Mason, Georgina Okecha, Matthew B. Reeves, John H. Sinclair, Mark R. Wills

**Affiliations:** ^1^ Cambridge Institute of Therapeutic Immunology and Infectious Disease and Department of Medicine, University of Cambridge School of Clinical Medicine, Cambridge, United Kingdom; ^2^ Institute of Immunity & Transplantation, University College London (UCL), London, United Kingdom

**Keywords:** human cytomegalovirus, latency, monocytes, reactivation, CD4+ T cells

## Abstract

Human cytomegalovirus (HCMV) infection is not cleared by the initial immune response but persists for the lifetime of the host, in part due to its ability to establish a latent infection in cells of the myeloid lineage. HCMV has been shown to manipulate the secretion of cellular proteins during both lytic and latent infection; with changes caused by latent infection mainly investigated in CD34+ progenitor cells. Whilst CD34+ cells are generally bone marrow resident, their derivative CD14+ monocytes migrate to the periphery where they briefly circulate until extravasation into tissue sites. We have analyzed the effect of HCMV latent infection on the secretome of CD14+ monocytes, identifying an upregulation of both CCL8 and CXCL10 chemokines in the CD14+ latency-associated secretome. Unlike CD34+ cells, the CD14+ latency-associated secretome did not induce migration of resting immune cell subsets but did induce migration of activated NK and T cells expressing CXCR3 in a CXCL10 dependent manner. As reported in CD34+ latent infection, the CD14+ latency-associated secretome also suppressed the anti-viral activity of stimulated CD4+ T cells. Surprisingly, however, co-culture of activated autologous CD4+ T cells with latently infected monocytes resulted in reactivation of HCMV at levels comparable to those observed using M-CSF and IL-1β cytokines. We propose that these events represent a potential strategy to enable HCMV reactivation and local dissemination of the virus at peripheral tissue sites.

## Introduction

A characteristic of human cytomegalovirus (HCMV), common to all the herpesviruses, is an ability to establish a lifelong latent infection. In healthy individuals, primary infection and subsequent reactivation of latent HCMV rarely cause disease; whereas in immunocompromised or immune suppressed patients, it can be life-threatening (1). Persistent HCMV infection is established in the immune competent despite a broad and robust immune response and this inability of the immune response to completely clear HCMV infection is likely due to the numerous immune evasion molecules encoded by the virus (2) as well as the ability of the virus to establish a latent infection. CD34+ progenitor cells and their monocyte derivatives are an established site of latent HCMV carriage *in vivo* (3–5) characterized by the carriage of viral genome in the absence of infectious virion production (3). However, viral gene transcription has been reported during latency (6–9) resulting in expression of numerous viral genes involved in the maintenance of viral latency, such as US28 (10–14). Carriage of HCMV in monocytes from the bone marrow to the peripheral tissue sites (15) can result in virus reactivation due to differentiation of monocytes to mature myeloid cells (16) and likely prolongs the lifespan of the infected monocyte (17). Evidence for the periodic subclinical reactivation of the virus has been surmised by the continual presence of large HCMV-specific T cell populations in infected individuals (18) and the suggested association of HCMV persistence with long-term illnesses such as vascular disease (19).

CD34+ progenitor cells – pluripotent cells that give rise to all circulating blood cells, populate the bone marrow environment (20). However, once the bone marrow resident CD34+ cell matures into monocyte derivatives, they migrate from the bone marrow to the peripheral blood (21) circulating for a day or so (22), patrolling the endothelial cell layer in an inactive state (23). The mature monocyte then either leaves the circulation to traffic to tissue sites where they may differentiate to, for instance, tissue resident macrophages (24, 25) or they die *via* apoptosis (22, 23, 26). In normal steady state conditions the type of mature myeloid cell the tissue resident monocyte differentiates into is dependent on signals from the local tissue microenvironment (20). Localized acute inflammation has been shown to recruit CD14+ monocytes in humans to the kidneys, intestine, skin, lungs and heart (25), inflammatory cytokines will provide the localized monocyte with very different signals to a bone marrow resident CD34+ cell. Latency and reactivation of HCMV is directly linked to the differentiation status of the infected cell. Latency is established in bone marrow resident CD34+ cells and the subsequent egress and terminal differentiation of CD34+ cells to macrophages and dendritic cells is concomitant with HCMV reactivation (27), reactivating HCMV has been identified in tissue resident macrophages *in vivo* (28). HCMV also manipulates the host’s cellular processes prolonging the life-span of monocyte cells (17, 26, 29) and promoting the migration of monocytes from the circulation into tissue sites (15). Therefore, it is important to improve our understanding of how latent HCMV infection manipulates the host’s cellular processes and immune responses in different tissue environments.

The manipulation of secreted cellular proteins (the cell secretome) by HCMV during lytic infection includes the release of factors that induce angiogenesis (30) and the release of inflammatory cytokines (31). We have previously shown that latent HCMV infection in CD34+ progenitors also modulates the cell secretome resulting in increased levels of CCL8, which recruits CD4+ T cells, as well as increased secretion of cellular IL-10 (cIL-10) and TGF-β, which suppress anti-viral functions of recruited CD4+ T cells (32). Another study of latent infection utilizing granulocyte macrophage progenitors have shown increased expression of CCL2, which enhances the migration of monocytes (33). A short-term model of latent infection in CD14+ monocytes revealed secretion of inflammatory immune mediators and promotion of differentiation to a macrophage-like phenotype (34). In a previous study, we also observed that viral IL-10 produced during latent infection of CD14+ monocytes results in upregulation of secretion of cIL-10 and CCL8 (35). However, a comprehensive assessment of the cellular secretome of latently infected monocytes has yet to be described and the effect of this latency-associated secretome on other immune cells has not been addressed.

Thus, using an established experimental model of HCMV latent infection in CD14+ monocytes (36), we have characterized the latency-associated changes in the cell secretome using chemokine and cytokine arrays. Consistent with previous studies in CD34+ and CD14+ cells (34, 35, 37), we observed upregulation of expression of cIL-10, CCL8 and CXCL10 by latently infected monocytes. We go onto show that the latency-associated secretome promoted the recruitment of immune cell subsets; in particular the recruitment of activated NK cells, CD8+ and CD4+ T cells *via* the interaction of CXCR3 expressed by the activated immune cells and CXCL10 present in the secretome. In addition, we also demonstrate that the latent secretome inhibited the production of anti-viral cytokines by stimulated CD4+ T cells. Intriguingly, the co-culture of activated CD4+ T cells with latently infected CD14+ monocytes promoted viral reactivation, likely due to the induction of differentiation pathways in the monocyte. Together, our data suggests that HCMV latently infected monocytes which have migrated to peripheral sites modulate the cellular secretome to enable reactivation but concomitantly prevent immune effector function to allow local dissemination of the virus in order to support long-term persistence of the viral infection of the host.

## Materials and Methods

### Donor Sample and Ethics Statement

Ethical approval for the work on healthy human samples was obtained from the Health Research Authority (HRA) Cambridge Central Research Ethics Committee (97/092) for this study, informed written consent was obtained from all healthy donors in accordance with the Declaration of Helsinki. Heparinized peripheral blood was collected from healthy donors or cells isolated from apheresis cones (National Health Service (NHS) Blood and Transplant Service). HCMV serostatus was determined using an IgG enzyme-linked immunosorbent assay (Trinity Biotech, Co. Wicklow, Ireland). 13 HCMV-seronegative, 7 HCMV-seropositive donors and 3 HCMV-seronegative apheresis cones were used in this study.

### Viruses

A low passage isolate of HCMV strain TB40/E and TB40/E UL32-GFP derived from it (a gift from Christian Sinzger, University of Ulm, Germany) and TB40/E-IE2-EYFP virus (a gift from Michael Winkler, Ulm University Hospital, Germany) were used for infections in this study, as indicated in the text. The infectious titer of the TB40/E strain was determined using HFFF cells; the pfu/ml (plaque forming units) was used to calculate the Multiplicity of Infection used to infect monocytes. The amount of TB40/E UL32-GFP and TB40/E-IE2-EYFP virus strains used to infect monocytes was assessed by titration of a range of concentrations of individual virus stocks on monocytes and choosing the input dose which resulted in a latent infection (relative absence of GFP or EYFP signals) compared to fluorescent cells following treatment with either PMA (Sigma Aldrich, Poole, UK) or M-CSF and IL-1β (Miltenyi Biotec, Bisley, UK), in order to reactivate the virus. Ultra-violet inactivation of virus strains used in this study was performed by placing an aliquot of virus in a tissue culture plate and placing this within 10cm of a UV germicidal (254nm) lamp for 60 minutes to inactivate the virus stock. We routinely test UV inactivated virus by infecting fibroblast and looking for IE protein expression, IE is not detectable by Immunofluorescence in these confirmatory studies.

### Preparation of Peripheral Blood Mononuclear Cells

Peripheral blood mononuclear cells (PBMC) were isolated from heparinized blood samples or apheresis cone mononuclear cells using either Lymphoprep (Axis-shield, Alere Ltd, Stockport, UK) or Histopaque-1077 (Sigma Aldrich) density gradient centrifugation.

### HCMV Latency and Infection of Monocytes

CD14+ Monocytes were isolated from donor PBMC by MACS using anti-CD14+ direct beads (Miltenyi Biotech), according to manufacturer’s instructions and separated on LS columns or an AutoMACS Pro (Miltenyi Biotec). Purified monocytes were adhered to a tissue culture plate at either 0.1 x 10^6^ cells per well density for 96 well plates, 0.3 x 10^6^ cells per well for 48 well plates or 0.5 x 10^6^ cells per well for 24 well plates, and then incubated overnight in X-VIVO 15 (Lonza, Slough, UK) supplemented with 2.5mM L-Glutamine (Sigma Aldrich) at 37°C in a humidified CO_2_ atmosphere.

Monocyte latent secretomes were generated by infecting adherent monocytes with TB40/E strain at a HFFF titrated MOI of 5 or the equivalent amount of UV-inactivated virus for 3 hours at 37°C in L-glutamine supplemented X-VIVO 15. Media was then replaced following a DPBS (Sigma Aldrich) wash and the infected cells were incubated in fresh supplemented X-VIVO 15 at 37°C in a humidified CO_2_ atmosphere. The supernatant (secretome) of the Mock, UV irradiated, and latently infected monocytes were collected and then replenished at days 3, 7 and 10 or 14. The collected supernatants were clarified by centrifugation. Latent infection was confirmed by harvesting RNA from the 3 cell treatments at day 7 and using RT-qPCR methods to compare relative expression of UL138 transcripts compared to the relative absence of IE72 transcripts controlled by GAPDH transcripts as explained in the supplementary methods with representative results also shown in **Figure S1**. We have demonstrated that in this experimental model of latency, that by day 7 latency is established, shown by expression of UL138 and absence of IE transcripts (38).

Latent infection of adherent monocytes in 96-well or 48-well plates with strains TB40/E UL32 GFP or TB40/E-IE2-EYFP at a pre-titrated concentration of virus (MOI was dependent on individual virus preparations) was performed for 3 hours in L-glutamine supplemented X-VIVO 15 at 37°C. Media was then replaced following a PBS wash and the infected cells were incubated in fresh supplemented X-VIVO 15 at 37°C in a humidified CO_2_ atmosphere for 4 – 6 days to allow latency to establish. At this time RNA was harvested from mock and infected cells to confirm latent infection by RT-qPCR and used for reactivation of HCMV from Latency experiments.

### Cytokine and Chemokine Array Analysis

**Figure 1:**
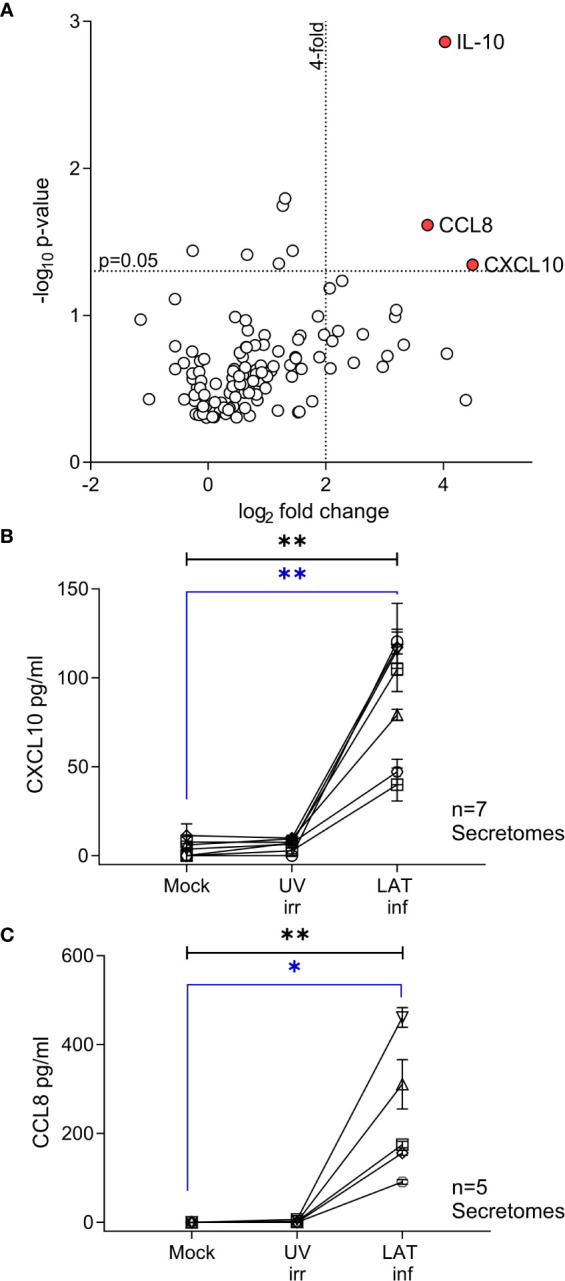
CXCL10, CCL8 and cIL-10 are upregulated in the secretome from latently infected CD14+ monocytes. A Volcano plot summarizing the protein array results from 3 independent day 7 secretomes, significant (p<0.05) highly upregulated (values > 4-fold) proteins are indicated by the red circle and label. Data was analyzed as described in the methods. **(A)** Confirmatory ELISA results for the production of CXCL10 **(B)** and CCL8 **(C)** show that both cytokines are significantly upregulated in the HCMV latent infected secretome at day 7. Seven and six independent secretomes were analyzed for CXCL10 and CCL8 production respectively. Friedman 1-way ANOVA results are shown in black on the graph; CXCL10 p=0.0012** and CCL8 p=0.0031**. Post hoc Dunn’s test results are shown in blue CXCL10 p=0.004** and CCL8 p=0.0133*.

The day 10 secretomes from mock, UV irradiated and Latent virus infected CD14+ monocytes were analyzed by Proteome Profiler Array – Human Chemokine Array Kit (R & D Systems, Abingdon, UK), RayBio Human Cytokine Array C1000 and RayBio Human Cytokine Array C5 (RayBiotech, supplied by Insight Biotechnology Ltd, Wembley, UK) following the manufacturer’s instructions. The arrays were imaged by autoradiography and then analyzed by ImageJ (Rasband, W.S., ImageJ, U. S. National Institutes of Health, Bethesda, Maryland, USA, https://imagej.nih.gov/ij/, 1997-2018.) to measure the density of each spot and to compare the relative amount of proteins expressed in different secretomes. The fold change in proteins expressed by the latent infected secretome were calculated as: $\;Fold\;change=\frac{\left(Density\;of\;Latent\;infection\;spot-Density\;of\;Mock\;infected\;spot\right)}{\left(Density\;of\;UV\;irradiated\;spot\;-Density\;of\;Mock\;infected\;spot\right)}$. Then whether the individual proteins in the array were significantly upregulated across all 3 experiments was tested by multiple student t-tests. The results of this analysis are presented as a volcano plot (39) in **Figure 1A**.

### Neutralization of Interferons During the Generation of Latent CD14+ Monocyte Secretomes

Adherent CD14+ monocytes were infected with mock, UV irradiated or TB40/E strain as described above in the presence of excess neutralizing anti-Human Interferon α [3µg; Clone: MMHA-6; EC50 20ng/ml (40)], anti-Human Interferon β [4µg/ml; Clone : MMHB-3 (41)] (both PBL Assay Science, USA) and Ultra-leaf anti-human Interferon γ [10µg/ml; Clone: B27 (42)] (BioLegend, London, UK) antibodies or Mouse IgG1 isotype control (Clone: 11711) (R & D Systems). The secretomes generated were harvested after 10 days, clarified and then analyzed by ELISA for CXCL10 and CCL8 cytokines.

### Generating Secretomes From Monocytes Treated With Recombinant Human IFNγ

Uninfected adherent monocytes were treated with recombinant human IFNγ protein (R&D Systems) over a concentration range of 100IU – 3.125IU for 4 hours. The IFNγ containing medium was removed and the cells washed with DPBS twice, then L-glutamine supplemented X-VIVO 15 was added to the treated cells and the cells were incubated at 37°C in a humidified CO_2_ atmosphere. Continuous application of 100IU/ml IFNγ was used as a positive control and X-VIVO 15 media alone was used as the negative control. Supernatants were harvested and media replenished on days 3, 6 and 10 and then analyzed by ELISA for CXCL10 production.

### Cytokine Quantification by Enzyme-Linked Immunosorbent Assay (ELISA)

Human IFNγ ELISA MAX Standard and Human CXCL10 and CCL8 ELISA MAX Deluxe sets (all Biolegend) were used to quantify cytokine concentrations in secretomes and supernatants. ELISAs were performed according to the manufacturer’s recommended protocols.

### Isolation of T Cells, B Cells, Monocytes and NK Cells

PBMCs were sorted into cellular subpopulations by positive selection using anti-CD14 microbeads to isolate monocytes, anti-CD4 microbeads for CD4+ T cells, anti-CD8 microbeads for CD8+ T cells and anti-CD19 microbeads for B cells with either LS columns or an AutoMACS Pro (Miltenyi Biotec). NK cells were isolated by either positive selection using anti-CD56 microbeads (Miltenyi Biotec) or negative selection using the NK cell isolation kit (Miltenyi Biotec) or with the EasySep Human NK cell enrichment kit (Stem Cell Technologies, Grenoble, France) following the manufacturer’s instructions.

### Preparation of Activated PBMC Subsets

Activated CD4+, CD8+ T and NK cells were generated in two ways. In the first method, isolated CD4+ and CD8+ T cells were re-suspended in RPMI-1640 (Sigma Aldrich) supplemented with 100IU/ml penicillin, 100µg/ml streptomycin and 10% Fetal Calf Serum (Gibco, Paisley, UK or PanBiotech, Wimborne, UK) – RPMI-10 and stimulated with irradiated (solid source γ-irradiator) autologous PBMC and 1µl/ml PHA (Sigma Aldrich) in the presence of 50IU/ml rhIL-2 (CFAR, NIBSC). The polyclonally activated T cell lines were maintained for up to 2 weeks at 37°C in a humidified CO_2_ atmosphere, with media and IL-2 replenishment every 5 days. Isolated NK cells were stimulated by an irradiated mixture of autologous PBMC and allogeneic lymphoblast cell line (BLCL) and 50IU/ml IL-2 in RPMI-10 and cultured for up to two weeks at 37°C in a humidified CO_2_ atmosphere, with periodic replenishment of media and IL-2. In the other method, total PBMC were stimulated with irradiated allogeneic PBMC and 50IU/ml rhIL-2 for the polyclonal activation of NK cells and the addition of 1µg/ml anti-CD3 (clone CD3-2) and 0.5µg/ml anti-CD28 (clone CD28-A) (both Mabtech AB, Nacka Strand, Sweden) for the polyclonal activation of T cells in RPMI-10. After 5 – 8 days stimulation the activated NK cells and CD4+ and CD8+ T cells were isolated by positive selection as described in section 2.8 or by using the NK cell, CD4+ T cell and CD8+ T cell isolation kits (Miltenyi Biotec) using an AutoMACS Pro, following manufacturer instructions. Activated NK cells and activated PBMC were sorted into two populations of cells (CXCR3+ and CXCR3-) using a BD FACSAria cell sorter by staining with Live Dead Far-Red (Thermo Fisher Scientific, Loughborough, UK) and CXCR3-PE (BioLegend).

### Transwell-Migration and CXCL10 Neutralization Assay

Transwell ChemoTx plates (5-µm pore size and 30-µl well volume) (Neuro Probe Inc, USA) were used to determine cell migration to latent and control secretomes. Cell subsets were fluorescently labelled using Calcein AM (BD Biosciences, Wokingham, UK) according to the manufacturer’s protocol. 2 x 10^4^ labelled cells in 20µl of X-VIVO-15 per well were transferred to the transwell plate and incubated at 37°C for 2 hours with supernatants from mock, UV and latently infected CD14+ monocytes in the lower chamber. Supernatants from monocyte-derived macrophages stimulated with LPS were used as a positive control, while X-VIVO-15 alone was used as a negative control. Migrated cells were enumerated using an UV microscope, five fields of view of each well were counted and all conditions were run in triplicate. CXCL10 neutralization assays were performed using supernatants or supernatants treated with anti-CXCL10 neutralizing antibodies or IgG2a isotype control (R & D Systems) for 1 hour using the recommended neutralization procedure and dose of the manufacturer, prior to being used in the migration assays.

### Flow Cytometry Methods

#### Phenotyping of Resting and Activated PBMC Subsets

The phenotype of resting and activated NK and T cell subsets was assessed by flow cytometry by staining with 3 antibody cocktails all containing Live Dead Far Red (Thermo Fisher Scientific); and (i) CD56 FITC, CXCR3 PE and CD3 PerCP Cy5.5; (ii) CD4 FITC, CXCR3 PE and CD3 PerCP Cy5.5; (iii) CD3 FITC, CXCR3 PE and CD8 PerCP Cy5.5 (details of antibody clones and manufacturer are listed in **Table S2**), following staining the cells were washed and fixed with 2% Paraformaldehyde in PBS solution (2% PFA (made from 4% PFA in PBS, Santa Cruz Biotechnology Inc, Dallas, USA)) and acquired on a BD Accuri C6 flow cytometer.

Further details of antibody cocktails used to assess the phenotype of resting and activated NK and T cell subsets are detailed in the supplementary methods (section 1.3 and **Table S2**), example gating analysis figures are also included (**Figure S2**).

#### Phenotyping of Monocytes and CXCR3+ and M-CSF Treated Co Cultured Monocytes

Latent infected monocytes and latent infected monocytes treated with either M-CSF and IL-1β or co-cultured with CXCR3+ T cells were harvested using Accutase (BioLegend). Details of the antibodies and methods used to analyze these samples can be found in the supplementary methods (Section 1.3, **Table S2** and **Figure S2**).

### HCMV Reactivation Experiments

Adherent monocytes were latently infected with either TB40/E-IE2-EYFP or TB40/E UL32-GFP strain of HCMV as described above. Between 4-days – 6-days infection the latently infected CD14+ monocytes were treated with either CXCR3+ sorted PBMC, activated CD8+, CD4+ T cells, NK cells, 20ng/ml M-CSF and 10ng/ml IL-1β (both Miltenyi Biotec) or PMA (Sigma Aldrich). The treated monocytes were observed by fluorescent microscope and EYFP or GFP expressing cells enumerated on the subsequent days post treatment.

To assess whether latently infected CD14+ monocytes treated with activated CD4+ T cells and M-CSF & IL-1β fully reactivated virus, fibroblasts were overlaid onto the treated monocytes and co-cultured for up to 14 days. Lytic HCMV infected fibroblasts were observed by fluorescent microscope and photographed. Quantification of HCMV DNA level in the overlaid fibroblast cultures was performed by isolating DNA using a previously described method (43), with quality and quantity being determined using a Nanodrop 1000 (Thermo Fisher Scientific), before HCMV genomic DNA (gDNA) level was determined using HCMV gDNA-specific primers (**Table S1**) with Luna Universal SYBR Green qPCR Master Mix (NEB, Hitchin, UK) as per manufacturer’s instructions on an ABI StepOnePlus (Thermo Fisher Scientific). DNA copy number was then determined by referencing to host GAPDH promoter copy number *via* the Pfaffl method (44).

### Suppression Assays and Cell Proliferation Assay

PBMC were depleted of CD8+ T cells by MACS using anti-CD8+ direct beads (Miltenyi Biotec), according to manufacturer’s instructions and separated on an AutoMACS Pro. The resulting CD4+ T cell & Antigen Presenting Cell (APC) PBMC were resuspended in either X-VIVO 15, X-VIVO 15 with 4ng/ml TGF-β and 10ng/ml IL-10 (both Miltenyi Biotec), neat Mock infected monocyte secretome, neat UV irradiated infected monocyte secretome or neat Latent Infected Monocyte secretome. The cells were then plated in 48-well tissue culture plates and incubated overnight at 37°C in a humidified CO_2_ atmosphere. After 24 hours incubation, the cells were stimulated with 1µg/ml anti-CD3 and 0.5µg/ml anti-CD28 (both Mabtech AB) and overlapping peptide pools for HCMV proteins (43) resulting in a 1:2 dilution of the secretomes and TGF-β/IL-10 mix. Following a further 24-hour incubation at 37°C in a humidified CO_2_ atmosphere, the plates were centrifuged, and supernatants harvested and then analyzed for the production of IFN-γ by ELISA. Full details of proliferation assays used to measure whether latent secretomes can suppress CD4+ T cells can be found in the supplementary material (Section 1.8).

### Statistics

Statistical analysis was performed using GraphPad Prism version 8.00 and 9.00 for Windows (GraphPad Software, San Diego, CA, USA). Multiple data sets groups were compared using a 1-way ANOVA Kruskall-Wallis test or Friedman test (for matched samples) with *post hoc* Dunn’s or Sidak’s multiple comparisons tests to correct for multiple testing false discovery.

## Results

### IL-10, CCL8 and CXCL10 Are Upregulated in the Latent HCMV Infected CD14+ Monocyte Secretome

We have previously shown that experimental latent HCMV infection of CD34+ progenitor cells alters the cellular secretome resulting in the upregulation of chemokines CCL8, CCL2 and secretion of TGF-β and cellular IL-10 (cIL-10) (32). Monocytes, which arise from CD34+ progenitor cells, are also a site of latent HCMV carriage *in vivo* (3). Consequently, we wanted to investigate whether the cellular secretome is also modulated in latently infected monocytes and, additionally, how this compares with the latency associated CD34+ secretome. Using an experimental model of latent HCMV infection of CD14+ monocytes (**Figure S1**), we screened secretomes from three independently generated latent HCMV infections of CD14+ monocytes using antibody arrays. In order to identify changes specific to latent infection, the fold change of cytokines in the secretome of latently infected monocytes was expressed relative to levels seen in the secretome of monocytes infected with UV-inactivated HCMV and corrected for background protein expression in mock infected monocytes (**Figure S3**). This analysis identified three proteins, IL-10, CCL8 and CXCL10, which were significantly upregulated (more than 4-fold) in all three latency-associated secretomes (**Figure 1A**). The production of CXCL10 and CCL8 by latently infected monocytes was confirmed by ELISA (**Figures 1B, C**); the level of both chemokines in the latently infected CD14+ monocytes were significantly increased over mock and UV inactivated infection controls.

**Figure 2:**
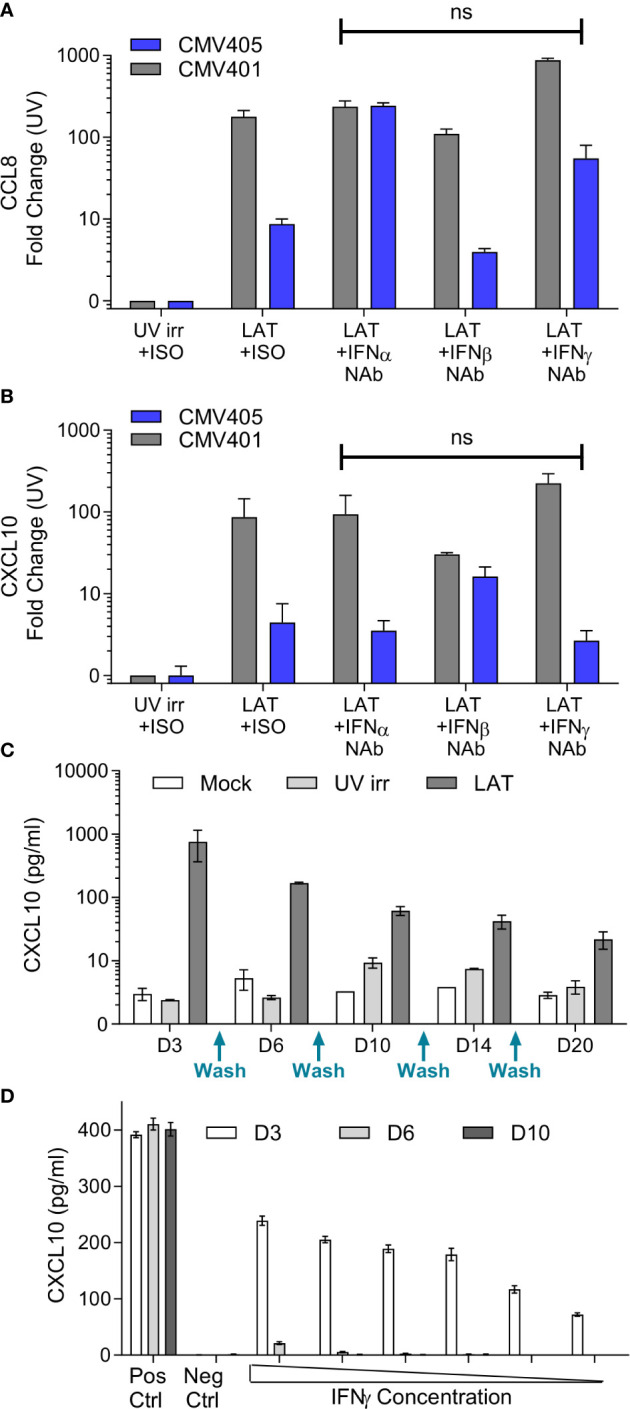
Production of CCL8 and CXCL10 by latently infected CD14+ monocytes is independent of interferon signaling. Neutralization of IFNα, IFNβ and IFNγ does not prevent the production of either CCL8 **(A)** or CXCL10 **(B)** from latent infected CD14+ cells at day 7 post infection. CXCL10 and CCL8 was measured by ELISA, with background chemokine production in response to mock infection subtracted and fold-change results above UV irradiated infection from 2 CMV sero-negative donors CMV401 and CMV405 shown. There was no significant (ns) change in the production of CCL8 or CXCL10 in the presence of neutralizing antibodies (Kruskall-Wallis test). **(C)** Freshly isolated monocytes were infected with TB40/E virus as described, cell supernatant was removed and replaced (indicated on x-axis) over a 20-day period, error bars represent SEM. This shows that CXCL10 is only reproduced by the latent infected monocytes over this time period. **(D)** Treatment of monocytes by recombinant IFNγ protein shows CXCL10 is detectable at day 3 but lost at later time points. Freshly isolated monocytes were stimulated with decreasing amount of IFNγ from 100IU/ml for 4 hours. Supernatants were collected on days 3 (white), 6 (grey) and 10 (black). Levels of CXCL10 were assayed using ELISA. Positive control consisted of continuous 100IU/ml IFNγ and negative control was media alone. This is the representative results of three repeats, error bars represent SEM.

The promoters of CXCL10 and CCL8 contain both Type I and II Interferon-responsive elements (45). As such, the overexpression of these chemokines could simply represent the induction of an anti-viral interferon response to infection rather than long term effects of latent carriage of virus. To determine if this was the case, latent secretomes were generated in the presence of neutralizing antibodies for IFN-α, IFN-β, IFN-γ or isotype controls. Analysis of the mock, UV irradiated and latency-associated secretomes by ELISA for CCL8 (**Figure 2A**) and CXCL10 (**Figure 2B**) shows that both chemokines are generated by the latently infected monocytes in the presence of interferon neutralizing antibodies and the magnitude of production is not significantly different. In addition, analysis of latency-associated secretomes after sequential replacement with fresh media across multiple time points (wash out experiments), revealed that latently infected monocytes continually produce CXCL10 (**Figure 2C**). In contrast, the treatment of monocytes with exogenous IFN-γ at the beginning of culture to stimulate CXCL10 production did not result in the continuous production of CXCL10 after IFN-γ is washed out (**Figure 2D**). Taken together, these data suggest that both CCL8 and CXCL10 are produced as a result of the latent HCMV infection of monocytes.

### Latency-Associated CD14+ Monocyte Secretomes Induce Activated CXCR3+ Immune Cell Migration Mediated by CXCL10

**Figure 3:**
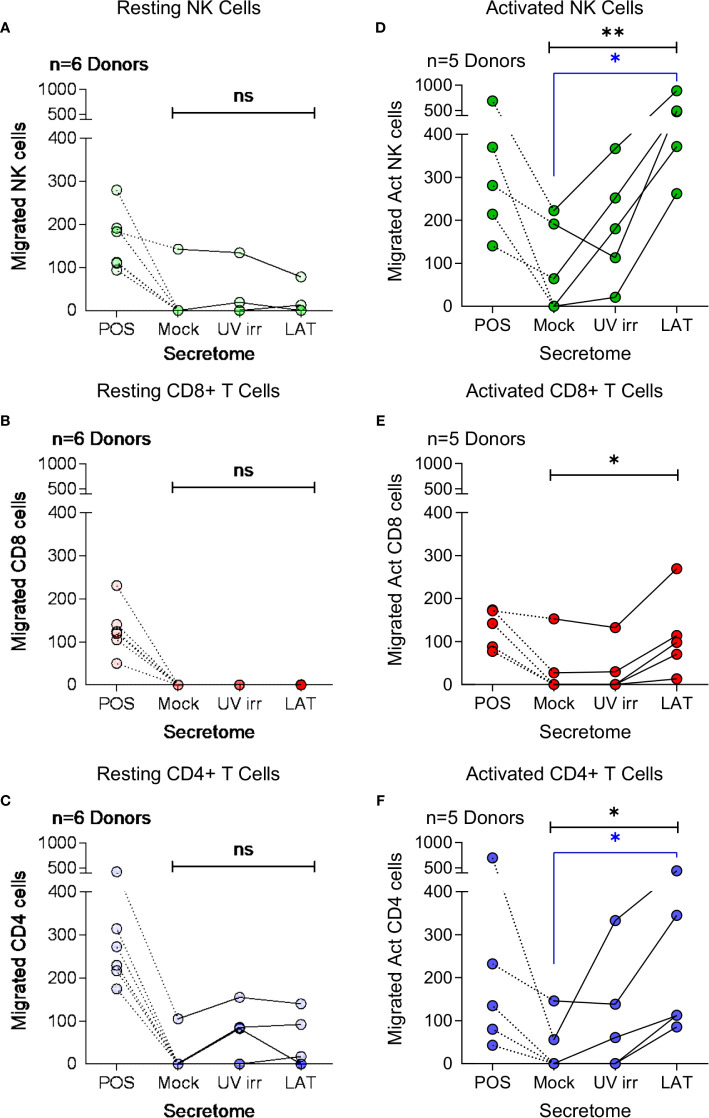
Migration of activated immune cell subsets in response to monocyte latent secretome. Transwell migration assays were performed in response to positive control, Mock, UV irradiated (UV irr) and Latent infected (LAT) CD14+ monocyte secretomes from day 7 harvest in multiple donors (indicated on each graph). Shown are the results from migration assays performed with resting NK cells – light green **(A)**, CD8+ T cells – light red **(B)** and CD4+ T cells – light blue **(C)** subsets. Following polyclonal activation (Act) transwell migration assays were performed with NK cells – dark green **(D)**, CD8+ T cells – dark red **(E)** and CD4+ T cells – dark blue **(F)**. All tested immune cell subsets from all donors migrated to the positive control, but there was only significant migration of the activated cell subsets (Friedman’s 1 way ANOVA – results indicated in black on graphs; Act NK p=0.0085**, Act CD8 p=0.0123* and Act CD4 p=0.0123*, non significant (ns) results are also indicated) to the latent infected secretomes. Activated NK cells and CD4+ T cells significantly migrated to the latent infected secretome (Dunn’s post-test shown in blue on graphs; NK p=0.0133* and CD4 p=0.0342*).

Cellular chemotaxis can be regulated by various chemokines and cytokines. In the context of HCMV infection, we have previously demonstrated that secreted factors from latently infected CD34+ cells promoted the migration of CD14+ monocytes and resting CD4+ T cells (32). Thus, we investigated the effect of latently infected CD14+ monocyte secretomes on cellular migration. Using a transwell migration assay, we assessed the impact of latency-associated CD14+ monocyte secretomes on the migration of NK cells (**Figures 3A, D**), CD8+ T cells (**Figures 3B, E**), CD4+ T cells (**Figures 3C, F**), B cells (**Figure S4A**), activated directly ex vivo or *in vitro*, and on monocytes (**Figure S4B**). In contrast to our observations with the latency-associated CD34+ secretome (32), we observed no significant migration of the freshly isolated lymphocyte cell subsets to the monocyte latency-associated secretome in six donors tested (**Figures 3A–C**; **Figures S4A, B**), despite the presence of CCL8 in these secretomes (**Figure 1C**). However, when cells were polyclonally activated prior to the assay, we saw significant migration of activated NK cells and CD4+ T cells (**Figures 3D, F**) and an upregulation of migration of activated CD8+ T cells (**Figure 3E**) to the latent infected monocyte secretomes.

**Figure 4:**
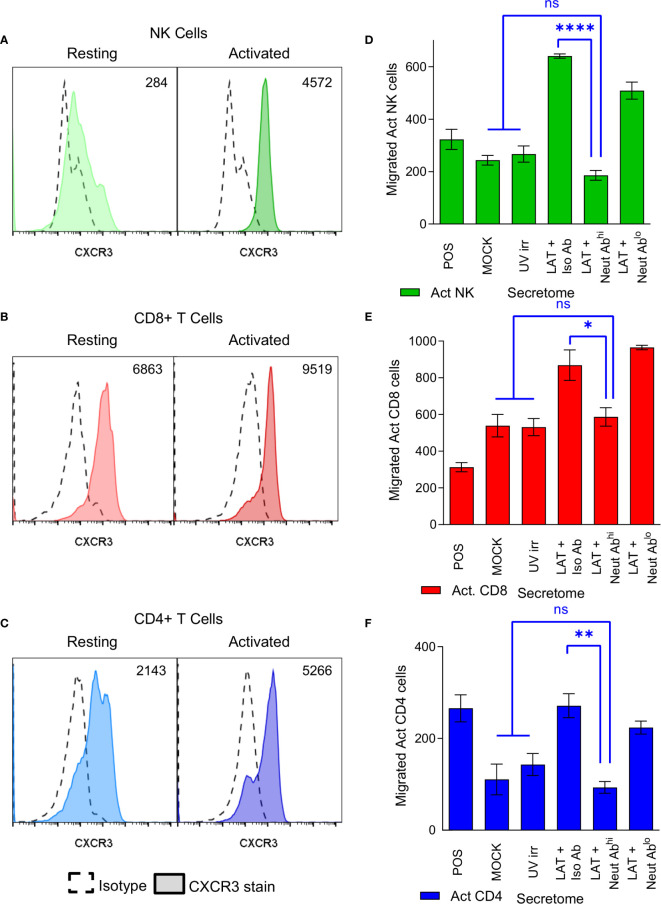
CXCR3 is upregulated on activated immune cell subsets and neutralization of CXCL10 abrogates migration of these cells. Histograms from flow cytometry analysis of CXCR3 expression on resting (left-hand histogram) and activated (right-hand histogram) NK cells **(A)**, CD8+ T cells **(B)** and CD4+ T cells **(C)** showing increased expression of CXCR3 on activated cell subsets (value shown on graph normalized geo-mean of CXCR3 expression for all cells analyzed). Representative results from 6 individual donors shown. Transwell migration assays were performed on the latent infected monocyte secretomes in the presence of CXCL10 neutralizing antibody or isotype controls showing that neutralization of CXCL10 significantly abrogates migration of activated NK cells **(D)**, CD8+ T cells **(E)** and CD4+ T cells **(F)** (1-way ANOVA with Sidak’s multiple comparison results shown in blue on the graphs; NK p<0.0001****, CD8 p=0.0488* and CD4 p=0.0032**, non-significant (ns) comparisons are also shown.).

CXCR3, a receptor that interacts with CXCL10 (46), is known to be upregulated on subsets of activated CD4+ T cells, CD8+ T cells and NK cells (47–49). We, therefore, analyzed the expression of CXCR3 on both resting and polyclonally activated T and NK cell subsets. The data show that there is low level expression of CXCR3 on all three subsets isolated directly *ex vivo* (**Figures 4A–C**, left hand histogram). However, CXCR3 expressed by un-activated T and NK cells has been shown to be non-responsive to its chemokine ligands (47), possibly explaining why *ex vivo* NK and T cells did not migrate to the latent secretome. The polyclonal activation of the NK cells, CD8+ and CD4+ T cells resulted in an upregulation of CXCR3 expression in all cases (**Figures 4A–C**, right hand histogram). Furthermore, flow sorting of activated NK cells into CXCR3+ and CXCR3- populations prior to performing a transwell migration assay showed that only the CXCR3 expressing cells had the capacity to migrate (**Figure S4C**). Importantly, antibody neutralization of CXCL10 present in the latently infected monocyte secretomes significantly abrogated the migration of all three activated cellular subsets (**Figures 4D–F**).

### Latent HCMV Infected CD14+ Monocyte Secretome Suppresses T Cell Function

**Figure 5:**
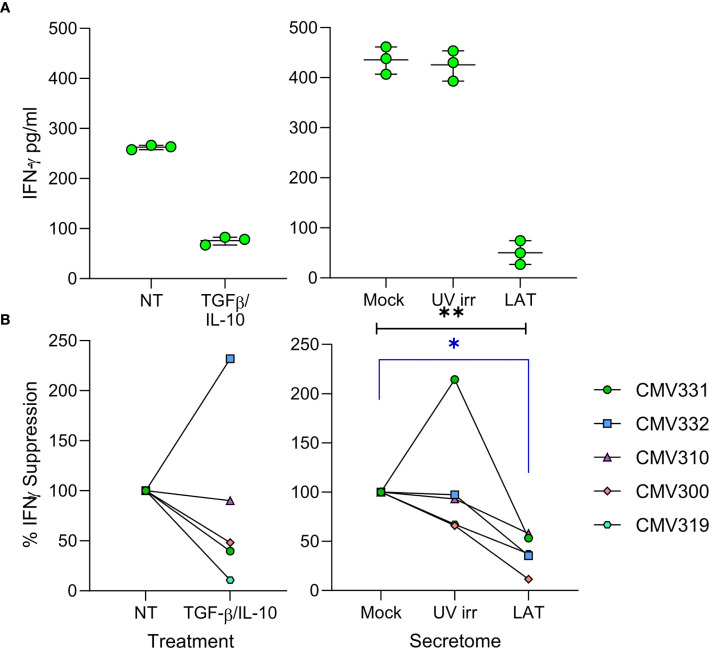
The latent infected monocyte secretome inhibits the production of IFNγ in response to polyclonal activation. CD4+ T cells were resuspended in either X-VIVO Media (Not Treated (NT)), media with added recombinant protein TGFβ & IL-10 (TGFβ/IL-10), Mock, UV irradiated (UV irr) or Latent infected (LAT) secretomes; following 24 hours pre-treatment the CD4+ T cells were stimulated with a mixture of anti-CD3 and anti-CD28 for a further 24 hours and then supernatants harvested. Production of IFNγ in the supernatants was measured by ELISA, representative results from one donor are shown **(A)**. Summary graphs showing the percentage suppression by either TGFβ/IL-10 treatment or the Latent infected monocyte secretomes of polyclonally stimulated CD4+ T cells from 5 donors **(B)**. The Latent infected secretomes significantly suppressed the production of IFNγ by polyclonal stimulation (Friedman matched 1-way ANOVA test (black line p=0.0085**) and Dunn’s corrected post-test (blue line p=0.0133*).

Virus driven recruitment of activated CD4+ T cells to latently infected cells does, at first, seem counter-intuitive with respect to virus survival; CD4+ T cells can be potently anti-viral and thus, hypothetically, if HCMV-specific, could limit HCMV reactivation (50, 51). However, we also observed elevated levels of cIL-10 (**Figure 1A**), an immunomodulatory cytokine that can suppress IFN-γ production by T cells (52), in the latently infected monocyte secretomes. Therefore, we hypothesized that the latently infected monocyte secretomes may also suppress possible anti-viral activity of the recruited CD4+ T cells. We assessed the production of IFN-γ by CD4+ T cells following polyclonal stimulation in the presence or absence of latency-associated secretomes. As expected, polyclonal stimulation of CD4+ T cells induced IFN-γ production (**Figure 5A**). However, this was significantly suppressed in the presence of the latently infected monocyte secretomes for each of five donors tested (**Figure 5B**, right-hand graph). Interestingly, treatment of stimulated CD4+ T cells with TGF-β and cIL-10 only suppressed IFN-γ production in three of the five same donors (**Figure 5B**, left-hand graph). Furthermore, we also observed a suppression of both IFN-γ production and cell proliferation by the latently infected monocyte secretome when the CD4+ cells were stimulated with HCMV antigen in some donors (**Figures S5A, B**).

To determine if the secretomes produced by latently infected cells could also suppress HCMV lytic replication, we utilized viral dissemination assays and show that spread of lytic virus was not inhibited (**Figures S6A, B**). We also determined if the secretomes caused an alteration in the phenotype of bystander uninfected monocytes (**Figures S6C, D**), however there was no change in expression of myeloid differentiation markers in monocytes incubated with latency-associated secretomes compared to secretomes from untreated monocytes. This evidence suggests that while proteins secreted by the latently infected monocyte recruit activated CD4+ T cells to its location, it can also suppress known anti-viral functions, such as production of IFN-γ, from these cells.

### CXCR3+ CD4+ T Cell Co-Culture Induces Reactivation From Latent HCMV Infected CD14+ Monocytes

**Figure 6:**
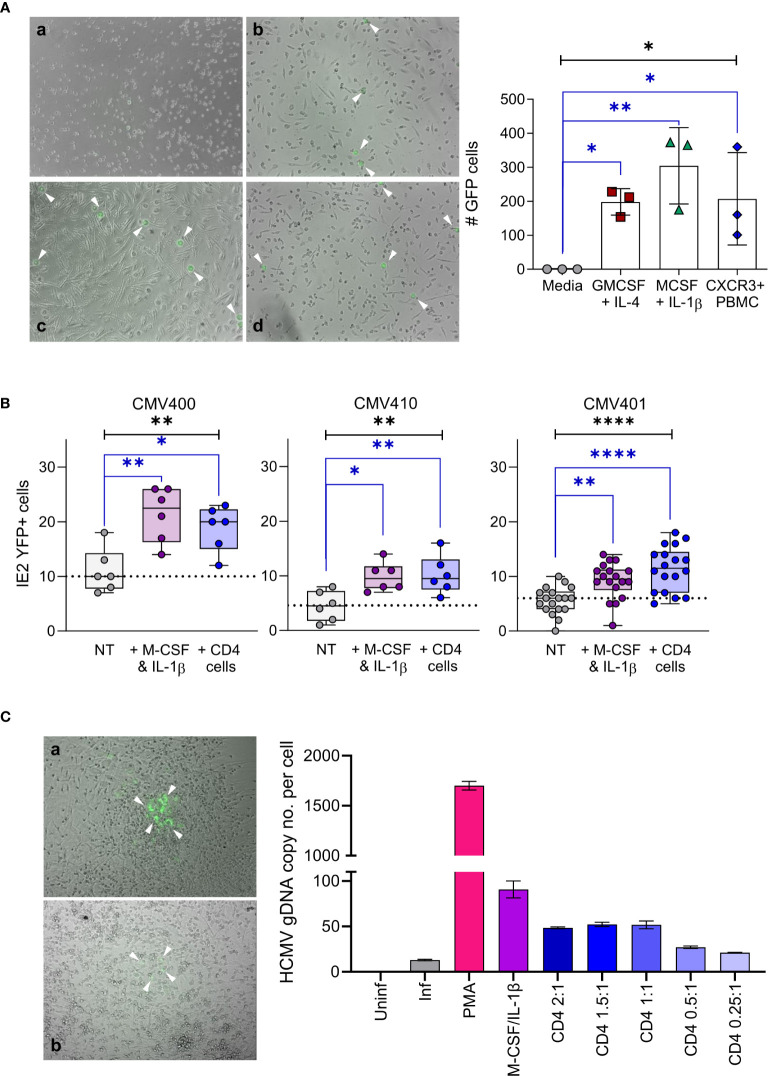
Activated CD4+ T cells can reactivate HCMV infection of latent infected monocytes. **(A)** CD14+ monocytes from a CMV sero-negative donor were infected with TB40/E UL32-GFP virus and co-cultured with activated PBMC or treated with cytokine cocktails (GM-CSF & IL-4 or M-CSF & IL-1β), virus reactivating GFP positive cells (white arrows) were visualized by microscope (right-hand panel – a Media, b M-CSF, c CXCR3+ PBMC & d GM-CSF) and enumerated (left-hand graph). All treatment conditions results in significant levels of GFP expressing cells representing reactivated virus (1-way ANOVA (black line: p=0.0112*) with corrected Dunnets post-test (blue lines: GM-CSF p=0.044*, M-CSF p=0.0048**, PBMC p=0.0363*). Shown is one representative donor of 4 different donors analyzed. **(B)** CD14+ monocytes latently infected with TB40/E IE2-YFP virus were co-cultured with activated CD4+ T cells or treated with M-CSF & IL-1β in 3 CMV sero-negative donors, reactivating YFP positive cells were enumerated. Both activated CD4+ T cells and M-CSF & IL-1β significantly increased reactivation of HCMV from latently infected autologous CD14+ monocytes (Kruskal Wallis 1-way ANOVA (Black line: CMV400 p=0.0049**; CMV410 p=0.0078**; CMV401 p<0.0001****) and Dunn’s post-test (Blue line CMV400 MCSF p=0.003**, CD4 p=0.03*; CMV410 M-CSF p=0.0133*, CD4 p=0.0098**; CMV401 MCSF p=0.0086**, CD4 p<0.0001****)). **(C)** CD14+ monocytes latently infected with TB40/E IE2-YFP virus were co-cultured with activated CD4+ T cells, treated with M-CSF & IL-1β or treated with PMA, then the cultures were overlaid with fibroblasts to measure the production of infectious virions. The resulting plaques were visualized by microscope (left-hand panel – a M-CSF & b Act CD4+ Treated) and IE2-YFP infected fibroblast plaques are indicated (white arrows). The infection of fibroblasts was quantified by measurement of genomic HCMV DNA present in each condition (right-hand graph), HCMV genome copy number was determined by qPCR of UL44 promoter relative to host GAPDH promoter. This demonstrates that detection of IE2-YFP positive monocytes can be used as a surrogate for the production of infectious virions in this system.

The accumulation of CXCL10 in the latently infected monocyte secretome and the consequent recruitment of activated lymphocyte cell subsets was unexpected (32). However, virally induced supernatants from monocytes that recruited these activated lymphocyte cell subsets also simultaneously reduced their effector function. Consequently, we reasoned that recruitment of activated immune cells to the site of latent infection (as long as their effector functions were suppressed) might, in some way, have a pro-viral effect on latency and/or reactivation. To interrogate this in more detail, we initially performed a co-culture experiment with CXCR3+ PBMC and monocytes latently infected with either TB40/E UL32-GFP or TB40/E IE2-YFP tagged strains of HCMV. Both pp150 (encoded by UL32) and IE2 proteins are expressed during lytic replication of the virus and, thus, can be used as markers of HCMV reactivation. Virus reactivation from latently infected monocytes was induced by culturing them in the presence of either GM-CSF and IL-4 or M-CSF and IL-1β cytokines which differentiate monocytes into a dendritic cell or macrophage like phenotype, respectively. When latently infected monocytes were incubated with CXCR3+ PBMCs, virus reactivation (UL32-GFP expression) was observed (**Figure 6A** left-hand panel) which was shown to be statistically significant (**Figure 6A** right-hand graph). Importantly, these levels of reactivation were comparable to that observed with the cytokine cocktails that promote dendritic (IL-4/GM-CSF) and macrophage (IL-1β/M-CSF) differentiation (**Figure 6A**). To determine which CXCR3 expressing cells could drive HCMV reactivation, we assessed the contribution of individual cell populations. A comparison of co-cultures of separate CXCR3+ populations of NK cells, CD8+ T cells and CD4+ T cells with latently infected monocytes alongside positive control for reactivation M-CSF and IL-1β treatment of infected monocytes was performed. The results show that co-culture with purified activated NK cells or CD8+ T cells did not result in virus reactivation in three separate donors tested (**Figure S7A**). Co-culture with activated NK cells may result in killing of latently infected monocytes, as the number of reactivating cells is lower than in the other conditions, this is not however a significant repression of infected monocyte numbers. In contrast, co-culture of activated CD4+ T cells with latently infected monocytes promoted virus reactivation at levels that were comparable to those observed following monocyte differentiation with M-CSF and IL-1β cytokines (**Figure 6B**). We also demonstrated that expression of IE2-YFP in monocytes, is indicative of the production of infectious virions, as the addition of fibroblasts to the reactivating monocyte culture results in infection of the fibroblast cell layer. Fibroblast overlaid on monocytes treated with M-CSF and IL-1β cytokines and co-cultured with CXCR3+ CD4+ T cells formed IE2-YFP positive infectious plaques (**Figure 6C** left-hand panel). This observation was quantified by the measurement of genomic HCMV DNA present in the fibroblast overlaid cultures (**Figure 6C** right-hand graph), showing the presence of HCMV DNA in all reactivating conditions (PMA and cytokine treated and activated CD4+ T cell co-culture).

Cytokines produced by allogeneically stimulated T cells have been demonstrated to promote virus reactivation in supernatant transfer experiments (53) and, thus, we asked whether cytokines produced by polyclonally stimulated CD4+ cells were similarly able to induce virus reactivation. Supernatants derived from activated immune cell subsets were co-cultured with a THP-1 monocytic cell line stably transfected with an integrated MIEP driven GFP expression cassette which act as a model of differentiation dependent induction of MIEP activity - when the THP-1 cells differentiate GFP is induced. Using this model cell line, we observed that supernatants derived from polyclonally activated CD4+ T cells do not promote increased MIEP expression whereas co-culture with activated CD4+ T cells do increase GFP expression (**Figure S7B**). This suggests that a physical interaction between the activated CD4+ T cell and monocyte is required. Phenotype analysis of monocytes co-cultured with CD4+ T cells showed that monocytes increased expression of T cell co-stimulation molecules CD80 and CD86 whereas monocytes differentiated with M-CSF and IL-1β increased expression of macrophage associated markers CD64 and CD68 (**Figures S8A, B**), all these upregulated markers are consistent with monocyte differentiation to myeloid derivatives. CD4+ T cell activation was confirmed by increased expression of CD40L, CXCR4 and 4-1BB alongside increased CXCR3 expression. In addition, MHC Class II (HLA-DR) was robustly upregulated on CXCR3+ CD4+ T cells in multiple donors (**Figure S8C**).

It has been reported that ligation of CD4 expressed on monocytes, by MHC Class II molecules expressed on other cells, promotes differentiation to macrophages (54) *via* Src family kinase (SFK), mitogen-activated protein kinase (MAPK) and extracellular signal-regulated kinase (ERK) pathways. Given the importance of these pathways in HCMV reactivation from dendritic cells (55, 56) we attempted to investigate whether inhibitors of ERK-MAPK signaling (U0126) and Src Family Kinases (PP2) prevented reactivation in our co-culture model. Unfortunately, treatment of latently infected monocytes with the inhibitors for the 96 hours incubation required for activated CD4+ T cells and M-CSF and IL-1β to trigger expression of IE2 protein was toxic. We investigated if by detecting IE72 mRNA by RT-qPCR we could perform these reactivation experiments over a shorter time period when these inhibitors would be less toxic to the cells. Latently infected monocytes were stimulated with either PMA (which induces rapid reactivation and IE72 expression) or M-CSF & IL-1β in the presence of U0126 inhibitor or its inactive control. The results at 24 hours post stimulation show PMA drives IE72 transcripts and this is partially inhibited by 10µm U0126 and not U0124 (the inactive analog), however M-CSF & IL-1β did not induce IE72 at this time point. By 48 hours PMA drive IE72 was no longer inhibited by U0126, as such inhibitions of these signaling pathways in an experimental set up that takes 96 hours to cause reactivation is not tractable.

## Discussion

Taken together, our analyses of the latency-associated secretome of monocytes is consistent with the view that latent HCMV infection results in modulation of the cellular secretome of the myeloid lineage which profoundly affects the latent cell microenvironment and modulates host immune responses to the latent reservoir (57, 58). Whilst carriage of latent HCMV genome by monocytes is likely to be short lived due to the limited lifespan of monocytes once they have migrated to the periphery (15, 22, 24), viral genomes can be detected in CD14+ monocytes isolated from healthy HCMV infected individuals (43, 59) and, importantly, the virus can be reactivated from these cells (3, 16, 53, 60). Therefore, consideration of the impact of latent infection on the local micro-environment in peripheral tissue sites, not just bone marrow sites of latency, is crucial for a full understanding of latency and reactivation *in vivo* and may be particularly helpful in the development of therapeutic measures to target HCMV reactivation in transplantation patients or pregnant women, the latter of which can lead to congenital HCMV (cCMV) sequalae in the new-born.

Previously, we have shown that experimental latent infection of CD34+ progenitor cells alters the cellular secretome to induce migration of CD4+ T cells and subsequent suppression of their effector function (32). That study revealed the impact of HCMV latent carriage on the CD34+ progenitor cellular microenvironment in the bone marrow but did not consider the very different environment encountered at peripheral tissue sites by the HCMV infected CD34+ myeloid derivatives. Here, we have shown that experimental latent infection of CD14+ monocytes, an established *in vitro* model system for latent infection *in vivo* (36), also results in changes to the cellular secretome causing upregulation of cIL-10, CCL8 and, in particular, CXCL10. Our analyses are consistent with another study of short-term latency in monocytes (34), but that study did not address the functional consequences of increases in latency-associated CCL8 and CXCL10 and did not identify an increase in cIL-10. These differences in latent secretome analyses may be attributable to the fact that, in our study, latency-associated secretomes were analysed at much later times (up to 14 days latency). The bioactivity of the CD14+ monocyte latent secretome differs to that observed for CD34+ progenitor cells in that monocytes and CD4+ T cells isolated directly *ex vivo* did not specifically migrate to the CD14+ secretome. Instead, we observed a significant recruitment of activated NK cells, CD8+ and CD4+ T cells, all with increased CXCR3 expression, to the CD14+ monocyte latency-associated secretome. Migration of these immune cell subsets was abrogated by neutralisation of CXCL10, a known ligand of CXCR3, in the CD14+ latency-associated secretome. Suggesting that the migration of activated CD8+ and CD4+ T cells as well as NK cells is mediated *via* interaction of CXCL10 present in the latently infected monocyte secretomes and that this effect is specific to CXCR3 expressing immune cells. Clear parallels from our study can be drawn with other infections, for example, the expression of CXCL10 was IFN independent – an observation also made with Hepatitis A infection (61). Furthermore, herpes simplex virus promotes CXCR3-mediated migration of CD4+ T cells to sites of infection (62) and CD8+ T cells to sites of latent infection (63). Recruitment of NK cells in the lung during Influenza A infection is similarly dependent on CXCR3 expression (64). These results could represent host driven anti-viral responses although, at least in the case of HCMV, the recruitment of CXCR3+ cells appears to be virus driven.

Activated antigen specific CD4+ T cells directed against HCMV have been demonstrated to be highly anti-viral (50, 51). Thus, it seems counterintuitive for the latent infection to modulate the cellular secretome to produce chemokines that attract activated and potentially antigen-specific immune cells to latently infected monocytes. However, our previous studies of HCMV latent infection in CD34+ progenitors showed that the latent secretome was also able to suppress inflammatory cytokine production by CD4+ T cells due to the presence of immunomodulatory cytokines including cIL-10 and TGF-β (32) as well as HCMV vIL-10 (35). This observation also holds true for the monocyte secretome, which we demonstrated has a significant inhibition on the production of IFNγ by CD4+ T cells following polyclonal activation. Whilst the suppression of activated CD4+ T cell anti-viral activity at the local site of latent monocytes would enable persistence of the virus infection, it does not entirely explain why activated immune cells are recruited. It may be that the secretion of CXCL10 and the recruitment of activated T cells is an unintended, and unwanted, consequence of the latent infection. Alternatively, the cellular secretome from latently infected monocyte might be promoting the recruitment of activated CD4+ T cells to the local tissue microenvironment for a pro-viral purpose.

While the establishment of cellular latency in reservoir sites such as CD34+ progenitor cells within the bone marrow is a long-term survival strategy for HCMV within the infected host, virus has to be able to reactivate in order for viral replication and dissemination to occur with a potential to transmit to a new host. In order to achieve this, the virus needs to be able to react to favourable changes in the cellular environment and external signals in order to initiate reactivation and provide a permissive cellular environment for virus replication. It is well established that HCMV reactivation is closely linked to myeloid cell differentiation and inflammatory environments (16, 53, 56, 65, 66). Utilisation of the monocyte, a derivative of CD34+ progenitor cells, to export the latent HCMV infection from the bone marrow to the periphery is one possible strategy for the virus to employ in order to reach an appropriate environment for virus replication. To overcome the short lifespan of monocytes in the periphery (15, 22, 24) the virus has been shown to manipulate cellular processes to promote a pro-survival state (17). Therefore, we hypothesized that the latent viral infection may be promoting a pro-inflammatory environment at peripheral sites by inducing production of CXCL10, rather than needing to relocate to a pre-existing inflammatory environment. The recruitment of activated CD4+ T cells by CXCL10 in the local environment may, then, trigger myeloid cell differentiation and successful virus reactivation, despite the concomitant recruitment of activated NK cells which may kill latently infected monocytes. Consistent with this, co-culture of latently infected monocytes with activated CD4+ T cells results in reactivation of virus and increased expression of myeloid differentiation markers. Further, our evidence suggests that direct interaction between the activated T cell and infected monocytes is required, possibly *via* ligation of HLA-DR, upregulated on CXCR3+ CD4+ T cells, with CD4 expressed on monocytes and other myeloid cell subsets (67). This CXCR3-mediated signalling to the monocyte has been shown to result in monocyte differentiation to a mature myeloid population (54) and is known to involve the ERK-MAPK pathway. Interestingly, the ERK-MAPK signalling pathway is also utilised by the M-CSF receptor (68) and Src family kinases are also implicated in reactivation of HCMV (55, 56). Unfortunately, due to the toxicity of the inhibitors to the ERK-MAPK signalling pathway over the time required to observe reactivation induced by both CD4+ T cell co-culture as well as M-CSF & IL-1β treatment we were not able to confirm this hypothesis. Other approaches to investigate the mechanisms involved in the reactivation of virus by activated CD4+ T cells include the use of neutralising antibodies to both CD4 (expressed by the monocytes) and HLA-DR (expressed by the T cells) or the use of recombinant HLA-DR to stimulate monocytes. These experimental approaches are limited to known ligand interactions, however, it is possible that reactivation of virus by activated CD4+ T cells or by differentiation with M-CSF and IL-1β employs similar signalling mechanisms with both resulting in monocyte differentiation to terminally differentiated myeloid cells.

Taken together, our observations support a model whereby latent HCMV promotes the recruitment of activated CD4+ T cells to monocyte sites of latency to promote viral reactivation which, *in vivo*, may support local dissemination of the virus at peripheral tissue sites. Importantly, HCMV simultaneously down-regulates effector functions of CD4+ T cells thereby “cherry picking” the effects of this CD4+ T cell recruitment by obtaining the benefit of T cell inflammation and contact-driven myeloid differentiation of the monocytes but preventing any T cell-mediated anti-viral immune response. In interpreting the evidence from this study it is important to note that this is an *ex vivo* model system, utilised because of the low frequency of latently infected monocytes in peripheral blood (69). There is evidence from many studies that subclinical reactivation of HCMV likely occurs in persistently infected, healthy, individuals; for instance there is a strong association of CMV infection with vascular disease in population studies (19) and CMV DNA has been identified in atherosclerotic plaques in a number of studies (70–72). It has been shown that HCMV latent infection results in profound changes in the latently infected cells besides just secreted cellular proteins - it also manipulates apoptotic pathways (17, 73) and modulates expression of cellular microRNAs (35, 74). HCMV latent infection of monocytes is also known to manipulate the recruitment of neutrophils (75) as well as the motility of latently infected monocytes to increase their trans endothelial migration (76). The association of persistent HCMV infection with vascular disease could also be partly explained by recruitment of activated T cells to peripheral sites of latency, as demonstrated here, as it is long established that CXCR3 expressing CD4+ T cells are recruited to atherosclerotic plaque sites (77). Therefore, the evidence from this study using an *ex vivo* model of latently infected monocytes, suggests potential avenues for future investigations looking for CMV reactivation in peripheral tissue sites in association with CXCL10 production and recruitment of CXCR3 expressing activated T cells.

In conclusion, the work presented here shows that latent HCMV carriage in monocytes at peripheral tissue sites results in manipulation of the cellular secretome of the infected cell in order to recruit activated immune cells resulting in reactivation and potential dissemination of the virus. This underscores how dynamic latent HCMV infection is and the extraordinary range of cellular processes that are manipulated by latent infection. We believe that identifying and understanding such latency-associated changes can only improve the development of therapeutic agents for use in clinical situations where CMV reactivation can cause significant morbidity and mortality such as in post-transplant or other immunosuppressed patients.

## Data Availability Statement

The protein array data summarised in **Figures 1** and **S2** of this study are publicly available. This data can be found here: https://www.ebi.ac.uk/biostudies/studies/S-BSST619.

## Ethics Statement

The studies involving human participants were reviewed and approved by Health Research Authority (HRA) Cambridge Central Research Ethics Committee (97/092). The patients/participants provided their written informed consent to participate in this study.

## Author Contributions

SJ, MW, JS, EP, IG and MR designed research. SJ, KC, IG, GS, AG, CH, EP, IM, GM, GO and MW performed research. SJ, KC, IG, GS, AG, IM, CH and MW analyzed data. SJ, MR and MW wrote the paper. All authors contributed to the article and approved the submitted version.

## Funding

This research was funded by the Medical Research Council (MRC: UKRI) grants MR/K021087, MR/S00081X/1 and MR/S00971X/1, and by Wellcome Trust collaborative grant 204870/Z/16/Z.

## Conflict of Interest

The authors declare that the research was conducted in the absence of any commercial or financial relationships that could be construed as a potential conflict of interest.
