# Supplementary material for: Latent Cytomegalovirus-Driven Recruitment of Activated CD4+ T Cells Promotes Virus Reactivation
Source: Front Immunol. 2021 Apr 12;12:657945. doi: 10.3389/fimmu.2021.657945 (PMC8072157; doi:10.3389/fimmu.2021.657945)
Supplement: Supplementary file 1 [file DataSheet_1.pdf]

Latent cytomegalovirus-driven recruitment of activated CD4+ T cells promotes virus reactivation

Sarah E. Jackson[1*†], Kevin C. Chen[1†], Ian J. Groves[1†], George X. Sedikides[1], Amar Gandhi[1], Charlotte J. Houldcroft[1], Emma L. Poole[1], Inmaculada Montanuy[1∞], Gavin M. Mason[1], Georgina Okecha[1], Matthew B. Reeves[2], John H. Sinclair[1] and Mark R. Wills[1*]

[1] Cambridge Institute of Therapeutic Immunology and Infectious Disease and Department of Medicine, University of Cambridge School of Clinical Medicine, Cambridge Biomedical Campus, Cambridge, CB2 2QQ, UK
[2] Institute of Immunity & Transplantation, UCL, Royal Free Hospital Campus, London, NW3 2PF, UK

[∞] Current address: Universidad Francisco de Vitoria, Ctra. Pozuelo-Majadahonda Km. 1.800, 28223. Madrid. Spain

[†] SEJ, KCC & IJG contributed equally to this work

*** Correspondence:**
Corresponding Authors Dr Sarah E. Jackson sej47@cam.ac.uk & Dr Mark R. Wills mrw1004@cam.ac.uk

Supplementary Material

1 Supplementary Materials and Methods

1.1 Viruses

A Merlin UL36-mCherry & UL32-GFP strain of HCMV (a gift from Richard Stanton, Cardiff University, UK), was used for infection of fibroblasts for use in a viral dissemination assay at a multiplicity of infection (MOI) of 0.1.

Latent infection was confirmed by harvesting RNA from the 3 cell treatments at day 7 and using RT-qPCR methods to compare relative expression of UL138 transcripts compared to the relative absence of IE72 transcripts controlled by GAPDH transcripts as described previously (1), representative results are shown in Fig. S1.

1.2 Molecular Biology analysis of HCMV latent infection, reactivation and lytic infection.

Latent infection was confirmed by harvesting RNA from the 3 cell treatments at day 7 using QIAzol from MiRNeasy Mini Kit (Qiagen, Manchester, UK), with quality and quantity of the RNA being determined using a Nanodrop 1000 (Thermo Fisher Scientific, Loughborough, UK). Taqman probe RT-qPCR was performed using QuantiTect Virus Kit (Qiagen) where cDNA is first synthesized before qPCR amplification, primers and probes used are detailed in table S1. Then the relative expression of UL138 transcripts was compared to the relative absence of IE72 transcripts controlled by GAPDH transcripts, as described previously (1), representative results are shown in Fig. S1B.

Previously, RT-PCR with end-stage PCR was performed to confirm latent infection, RNA was isolated from latent infected monocytes using RLT buffer and RNeasy Mini Kit (Qiagen). Reverse transcription of RNA was carried out from total RNA using a Promega reverse transcription kit and amplified using a 2x PCR Red Mix (Bioline) using parameters that have been described previously (2) and sequences are also included in table S1. Results from this analysis are shown in Fig. S1A.

Alternatively, RNA was isolated from plated CD14+ monocytes using RLT buffer and RNeasy Mini Kit (Qiagen), with quality and quantity being determined using a Nanodrop 1000 (Thermo Fisher Scientific), before elimination of genomic DNA and cDNA synthesis using 250ng of RNA with the Quantitect Reverse Transcription Kit (Qiagen). Relative transcript levels were then determined using HCMV cDNA-specific primers (Table S1) with Luna Universal SYBR Green qPCR Master Mix (NEB) as per manufacturer’s instructions on an ABI StepOnePlus. Transcript levels were normalised for primer efficiency and referenced to host GAPDH transcript level using the Pfaffl method (3).

Table S1. Primers and probes used in RT-PCR and qPCR assays

Oligo Name	Oligo Sequence (5’ to 3’)	Protocol
GAPDH sense	GAGTCAACGGATTTGGTCGT	RT-PCR
GAPDH antisense	TTGATTTTGGAGGGATCTCG	
UL138 sense	TGCGCATGTTTCTGAGCTC	RT-PCR
UL138 antisense	ACGGGTTTCACAGATCGAC	
IE sense	GGACCCTGTAATCCTGACG	RT-PCR
IE antisense	ATCTTTCTCGGGGTTCTCGT	

GAPDH sense	GGAAGCTTGTCATCAATG	RT-qPCR Taqman
GAPDH antisense	CCCCACTTGATTTTGGAG	
GAPDH probe	JOE-ATCACCATCTTCCAGGAGCGAG-BHQ1	
UL138 sense	CGCTGTTTCTCTGGTTAG	RT-qPCR Taqman
UL138 antisense	CAGACGATACCGTTTCTC	
UL138 probe	TAMRA-CCGACGACGAAGACGATGAAC-BHQ2	
IE72 sense	CAAGAACTCAGCCTTCCCTAAGAC	RT-qPCR Taqman
IE72 antisense	TGAGGCAAGTTCTCGAATGC	
IE72 probe	6FAM-CCAATGGCTGCAGTCAGGCCATG-BHQ1	
GAPDH sense	TGCACCACCAACTGCTTAGC	RT-qPCR - SYBR
GAPDH antisense	GGCATGGACTGTGGTCATGAG	
IE72 sense	GTCCTGACAGAACTCGTCAAA	RT-qPCR - SYBR
IEexon4 antisense	TAAAGGCGCCAGTGAATTTTTCTTC	
UL138 sense	ACGACGAAGACGATGAACCC	RT-qPCR - SYBR
UL138 antisense	CCCGATGAGATCTTGGTCCG	
GAPDH promoter sense	CGGCTACTAGCGGTTTTACG	Experimental HCMV gDNA quantification
GAPDH promoter antisense	AAGAAGATGCGGCTGACTGT	
UL44 promoter sense	AACCTGAGCGTGTTTGTG	Experimental HCMV gDNA quantification
UL44 promoter antisense	CGTGCAAGTCTCGACTAAG	

Conditions for each RT-PCR, RT-qPCR and gDNA quantification can be found in detail in the Poole *et al* Chapter in Human Cytomegaloviruses: Methods and Protocols 2nd Edition (4, 5)

1.3 Flow cytometry analysis

Full details of antibodies and reagents used in all flow cytometry experiments in this paper and supplementary material are listed in table S2.

Phenotyping of resting and activated PBMC subsets

The phenotype of resting and activated NK and T cell subsets was assessed by flow cytometry by staining with 3 antibody cocktails all containing Live Dead Far Red (Thermo Fisher Scientific); and (i) CD56 FITC, CXCR3 PE and CD3 PerCP Cy5.5; (ii) CD4 FITC, CXCR3 PE and CD3 PerCP Cy5.5; (iii) CD3 FITC, CXCR3 PE and CD8 PerCP Cy5.5, following staining the cells were washed and fixed with 2% Paraformaldehyde in PBS solution (2% PFA (4% PFA in PBS, Santa Cruz Biotechnology Inc)) and acquired on a BD Accuri C6 flowcytometer (BD Biosciences, Wokingham, UK).

Resting and activated NK and T cell subsets were also stained with an antibody cocktail containing; Live Dead Aqua (Thermo Fisher Scientific), CD3 Brilliant Violet 650, CD4 Brilliant Violet 605, CD8 Brilliant Violet 570, CD56 Brilliant Violet 785 and CXCR3 PE. Following staining the cells were washed and fixed with 2% PFA. The expression of activation markers on CXCR3+ CD4+ T cells was assessed by flow cytometry with the antibody cocktail detailed above plus the following; HLA-DR PE-Cy5, CD40L PerCP-Cy5.5, CXCR4 PE-Cy7 and 4-1BB APC. All Samples were acquired on a

LSR Fortessa (BD Biosciences) along with Fluorescence Minus One (FMO) controls using FACS Diva software (BD Biosciences). Samples were then analyzed using FlowJo software (Treestar, Oregon, USA). The gating sequence employed for analysis of these samples is illustrated in Fig. S2A.

Phenotyping of monocytes and treated co-cultured monocytes

Monocytes were treated with latent secretomes to assess whether there was any bystander effects on the phenotype of uninfected monocytes. Latent infected monocytes and latent infected monocytes were treated with either M-CSF and IL-1β or co-cultured with CXCR3+ T cells to assess whether these cells differentiated. Monocytes from both experiments were harvested from the tissue culture plates using Accutase (BioLegend, London, UK) according to the manufacturer's instructions. Harvested cells were washed in DPBS (Sigma) and then blocked with 1/50 Normal mouse serum (Thermo Fisher Scientific) and TruStain FcX (BioLegend) at manufacturer's recommended levels for 10 minutes at room temperature. Each sample was then split and stained with the some of the following pre-titrated antibodies: True-Stain Monocyte Blocker (BioLegend), Live Dead Aqua, HLA-DR Brilliant Violet 421, CD14 PerCP-Cy5.5, CD80 PE-Cy5, CD86 PE-Cy7, CD209 APC, HLA-ABC AxF-700, CD64 PE-Dz-594 and CD68 APC-Cy7 or True-Stain Monocyte Blocker, Live Dead Aqua, and appropriate fluorochrome conjugated isotype antibodies, for 30 minutes at 4ºC. Cells were washed in excess DPBS and then fixed with a 2% PFA solution. Samples were kept in the dark at 4ºC until acquisition on LSR Fortessa using FACS Diva software. Samples were then analyzed using FlowJo, and the gating strategy employed is illustrated in Fig. S2B, this phenotype panel was optimized using Fluorescence Minus One controls in addition to the matching isotype control staining for each sample.

Table S2. Antibodies used for Flow cytometry analysis of myeloid, NK and T cells

Antigen	Fluorochrome	Clone	Isotype	Cat. No.	Supplier
CD3	FITC	UCHT1	IgG1	300406	BioLegend
CD3	PerCP-Cy5.5	UCHT1	IgG1	300430	BioLegend
CD3	BV650	OKT3	IgG2a	317324	BioLegend
CD4	FITC	RPA-T4	IgG1	300506	BioLegend
CD4	BV605	OKT4	IgG2b	317438	BioLegend
CD8a	PerCP-Cy5.5	RPA-T8	IgG1	301032	BioLegend
CD8a	BV570	RPA-T8	IgG1	301038	BioLegend
CD14	PerCP-Cy5.5	M5E2	IgG2a	301824	BioLegend
CD56	FITC	HCD56	IgG1	300406	BioLegend
CD56	BV785	5.1H11	IgG1	362550	BioLegend
CD64	PE-Dz594	10.1	IgG1	305032	BioLegend
CD68	APC-Cy7	Y1/82A	IgG2b	333822	BioLegend
CD80	PE-Cy5	2D10	IgG1	305210	BioLegend
CD86	PE-Cy7	IT2.2	IgG2b	305422	BioLegend
CD137 (41BB)	APC	4B4-1	IgG1	309810	BioLegend
CD154 (CD40L)	PerCP-Cy5.5	24-31	IgG1	310834	BioLegend
CD209 (DC-SIGN)	APC	9E9A8	IgG2a	330108	BioLegend
CXCR3	PE	G025H7	IgG1	353706	BioLegend
CXCR4	PE-Cy7	12G5	IgG2a	306514	BioLegend
HLA-ABC	AxF-700	W6/32	IgG2a	311438	BioLegend

HLA-DR	PE-Cy5	L243	IgG2a	307608	BioLegend
HLA-DR	BV421	L243	IgG2a	307636	BioLegend
IgG1	PE-Dz594	MOPC-21		400176	BioLegend
IgG1	PE-Cy5	MOPC-21		400118	BioLegend
IgG2a	BV421	MOPC-173		400260	BioLegend
IgG2a	PerCP-Cy5.5	MOPC-173		400251	BioLegend
IgG2a	APC	MOPC-173		400222	BioLegend
IgG2a	AxF-700	MOPC-173		400248	BioLegend
IgG2b	PE-Cy7	MPC-11		400326	BioLegend
IgG2b	APC-Cy7	MPC-11		400328	BioLegend

Abbrv: BV = Brilliant Violet; Dz = Dazzle; AxF = Alexa Fluor.

1.4 HCMV Reactivation experiments with inhibitors

Adherent monocytes were latently infected with either TB40e-IE2-EYFP or TB40\e UL32-GFP strain of HCMV as described above. Between 4-days – 6-days infection the latently infected CD14+ monocytes were treated with either CXCR3+ sorted PBMC, activated CD8+, CD4+ T cells, NK cells, 20ng/ml M-CSF and 10ng/ml IL-1β (both Miltenyi Biotec) or PMA (Sigma Aldrich) in the presence of MEK/ERK inhibitor U0126 or the inactive analog U0124 (Calbiochem, both 10µM) or Src family kinase inhibitor PP2 (Sigma Aldrich, 20nM). To assess whether treatment of monocytes with these inhibitors prevented reactivation of the virus, production of IE RNA transcripts was assessed using RT-qPCR SYBR quantification as detailed in section 1.2.

1.5 Cell proliferation assay

To measure proliferation of the CD4+ T cells following treatment with the latent secretomes, the CD4+ T cell & APC PBMC were labelled with Cell Trace Far Red proliferation kit for flow cytometry (Thermo Fisher Scientific), according to manufacturer’s instructions. Cells were then resuspended in either X-VIVO 15, neat Mock infected monocyte secretome, neat UV irradiated infected monocyte secretome, neat Latent Infected Monocyte secretome or X-VIVO 15 with TGF-β & IL-10, plated in round bottom 96 well plates and incubated overnight at 37°C in a humidified CO_2 atmosphere. After 24 hours incubation the cells were stimulated with anti-Biotin MACSiBeads particles loaded with biotinylated anti-human CD2, CD3, CD28 and CD137 (all Miltenyi Biotec) following manufacturer’s instructions, at a bead to cell ratio of 1:2 or HCMV protein peptide pools and incubated for a further 6 days at 37°C in a humidified CO_2 atmosphere. Post incubation cells were harvested and washed in PBS prior to staining with Live Dead Green (Invitrogen), CD4-PE and CD3-PerCP-Cy5.5 (both BioLegend); cells were washed in excess DPBS and then fixed with a 2% PFA solution. Samples were then acquired on a BD Accuri C6 plus flow cytometer and the cell proliferation data analysed by FlowJo software.

1.6 Viral dissemination assay with supernatants

HFFFs were seeded in a 96-well plate to be 80 to 90% confluent when they were infected with HCMV Merlin UL36-mCherry & UL32-GFP strain. The next day Mock infected monocyte secretome, UV irradiated infected monocyte secretome, Latent Infected Monocyte secretome and a

positive control secretome (generated from PBMC stimulated with anti-CD3 and anti-CD28 antibodies) serially diluted were added to the assay. The viral dissemination assay was incubated at 37°C plus 5% CO_2, and viral dissemination was assessed at 9 days by detection of mCherry and GFP expression by flow cytometry. Samples were acquired using an Attune NxT (Thermo Fisher) and analysed by FlowJo software.

1.7 THP-1-MIEP-eGFP Cell Line Experiment

THP-1-MIEP-eGFP cells (a gift from M. Van Loock, Johnson & Johnson, from which an isolated integrated HCMV MIEP expresses enhanced GFP upon differentiation, were grown in RPMI 1640 medium (Sigma Aldrich) supplemented with 20% heat-inactivated foetal bovine serum (FBS) (PAN Biotech), penicillin (100U/ml) and streptomycin (100µg/ml) (Sigma Aldrich) and incubated at 37°C in a 5% CO_2 environment. The THP-1-MIEP-eGFP cell line was co-cultured with either LPS (a positive control to induce differentiation of the THP-1-MIEP cells), supernatants derived from activated NK, CD8+ T, CD4+ T or CXCR3+ cells or activated cell subsets for 7 days and then GFP expression in the cells was measured using the BD Accuri C6 plus flow cytometer and data was analyzed by FlowJo software.

2 Supplementary Figures

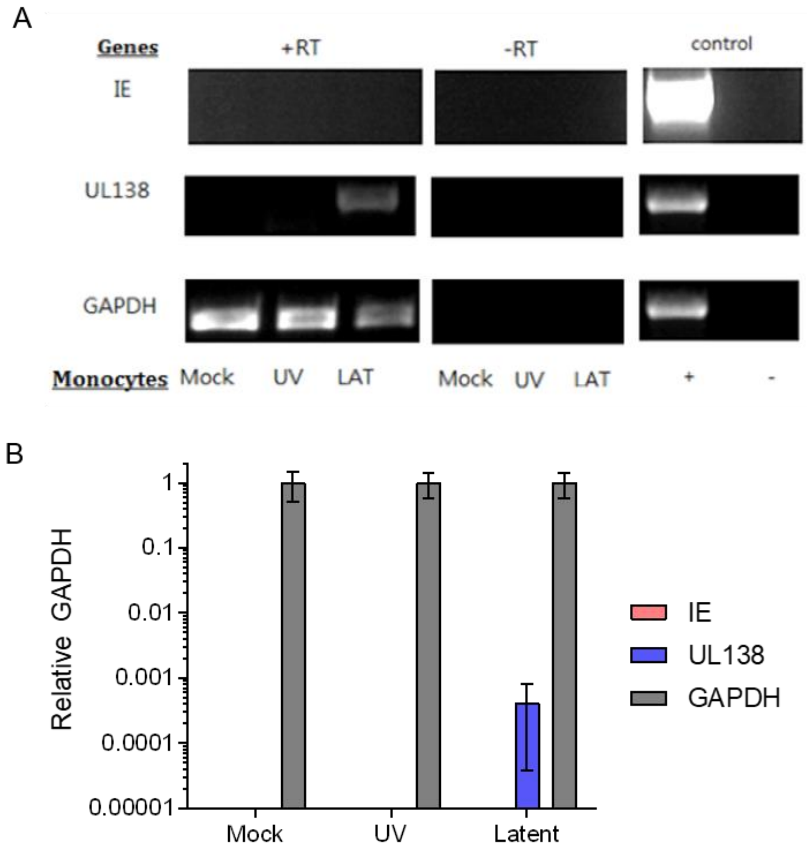

Figure S1 - RT PCR and RT qPCR results from Experimental Latent infections

RNA was extracted from Mock, UV irradiated and Latent infected monocytes at day 10 post infection and RT-PCR (A) was performed to detect the presence of Immediate Early (IE) (expressed in lytic infection), gene UL138 (expressed in latent and lytic infection) and GAPDH transcripts. HFF lytically infected with the same strain of HCMV were also analyzed as a positive control. Alternatively, RNA was extracted from samples at day 7 post infection and RT-qPCR for IE, UL138 and GAPDH was performed using Taqman probe method, graph shows expression of each gene relative to GAPDH and indicates that only UL138 expression was detectable in the latent infected sample (B).

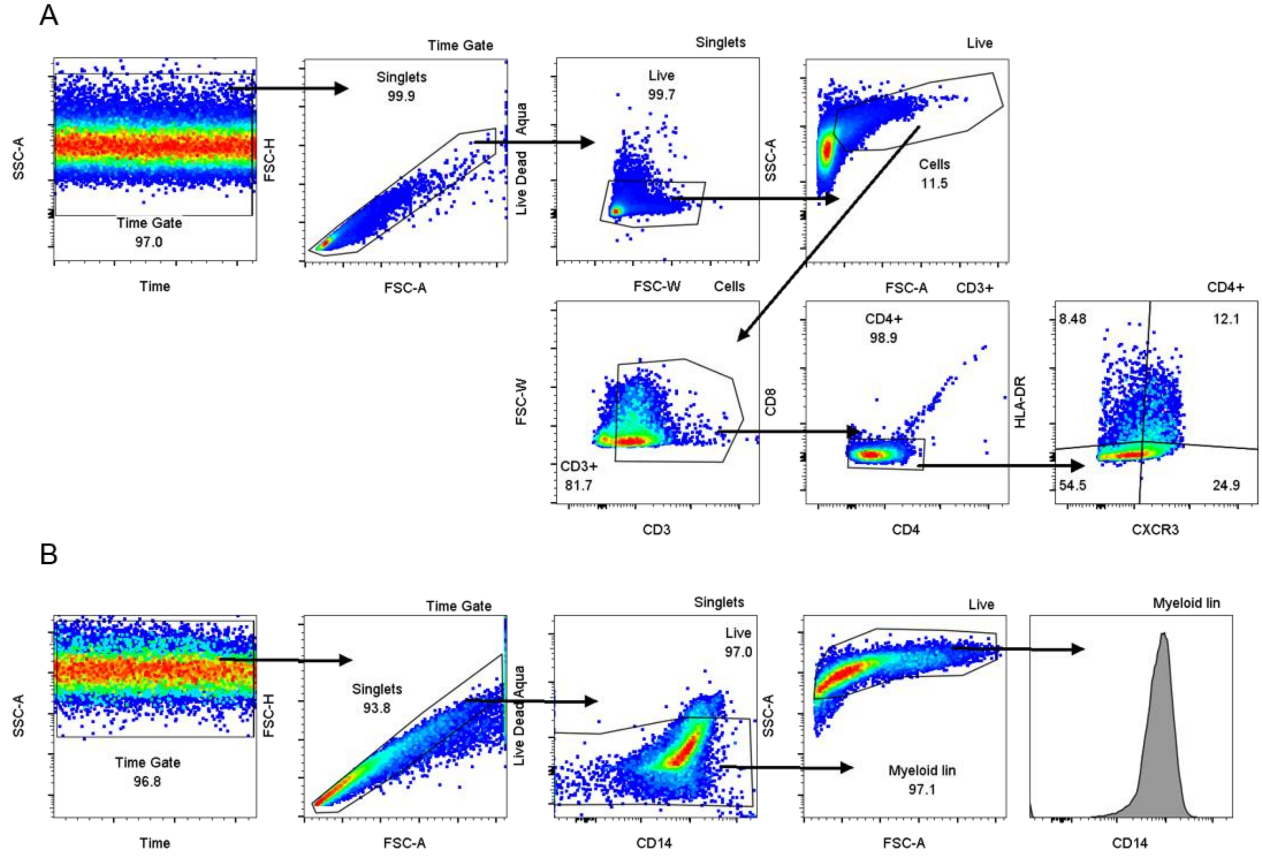

Figure S2 – Flow Cytometry gating schemes

(A) Activated CD4+ T cells were analyzed for expression of activation markers by flow cytometry. Representative plots from 1 donor are shown illustrating the gating strategy for analyzing these samples using FlowJo. First a Time vs Side scatter gate was drawn, to identify the main flow of cells, then these cells were gated for single cells (Forward scatter area vs Forward scatter height), live cells (forward scatter width vs Live Dead Aqua dye), then activated lymphocytes were gated (Forward scatter area vs side scatter area (log scale)). CD3 positive cells were identified (CD3 BV650 vs Forward Scatter width) and then CD4 positive cells gated (CD4 BV605 vs CD8 BV570), the expression of CXCR3 and other activation markers were analyzed from this gate (illustrated CXCR3 PE vs HLA-DR PE-Cy5). The gating strategy for this phenotype panel was optimized using Fluorescence Minus One controls in addition to the matching isotype control staining for each sample.

(B) Monocytes from the bystander effect experiment or differentiation experiments following co-culture with either cytokines or CXCR3+ CD4+ T cells were analyzed for expression of a range of myeloid and myeloid differentiation markers. Samples were then analyzed using FlowJo, by using first a Time vs Side scatter gate, to identify the main flow of cells, then these cells were gated for single cells (Forward scatter area vs Forward scatter height), live cells (forward scatter width vs Live Dead Aqua dye), then monocyte/myeloid cells gate (Forward scatter area vs side scatter area (log scale)). Phenotype markers were analyzed from this gate. The gating strategy for this phenotype panel was optimized using Fluorescence Minus One controls in addition to the matching isotype control staining for each sample.

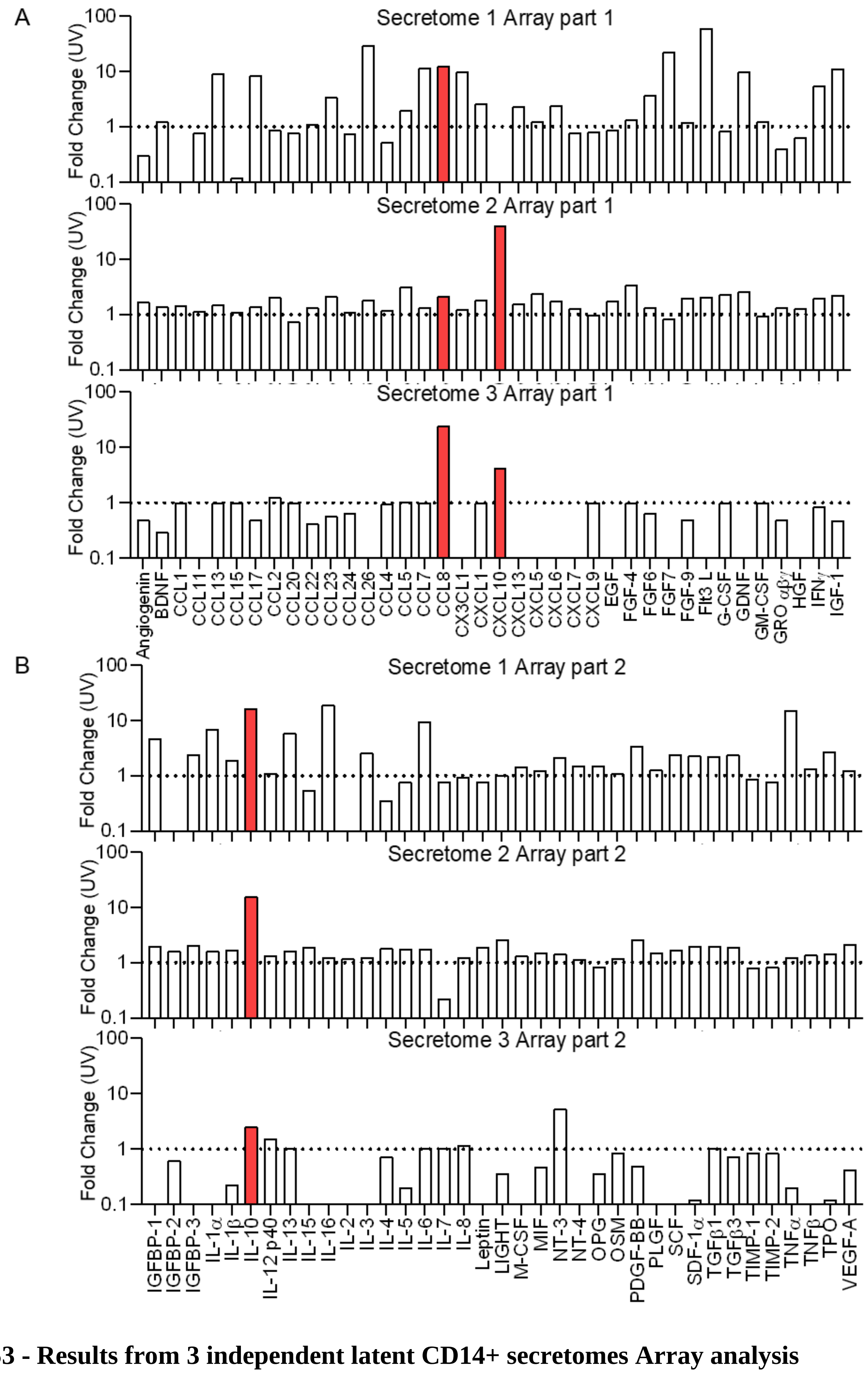

Figure S3 - Results from 3 independent latent CD14+ secretomes Array analysis

Presented are the summary results for Fold change of 75 proteins analyzed in the Latent infected secretome over the UV irradiated secretome following correction for background protein expression

in mock infected secretomes. (A) shows proteins for part 1 of the 75 proteins analyzed and (B) shows proteins for part 2 of the 75 proteins analyzed for all 3 independently generated secretomes. Significantly upregulated proteins presented in the volcano analysis (Figure 1) are highlighted as red bars in this data.

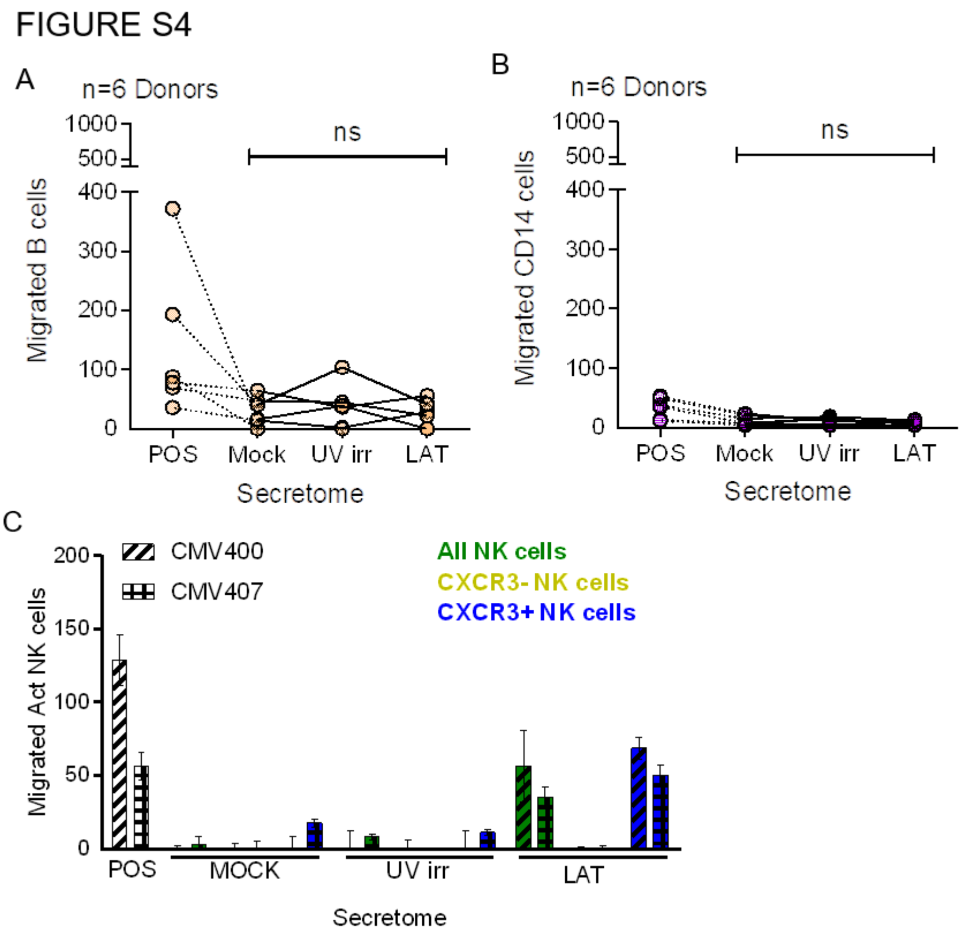

Figure S4 - Migration of B cells and Monocytes and sorted CXCR3 expressing NK cell subsets

Transwell migration assays to positive control, Mock, UV irradiated (UV irr) and Latent infected (LAT) CD14+ monocyte secretomes in multiple donors were performed with resting B cells – light orange (A) and CD14+ Monocytes – light pink (B). Both immune cell subsets migrated to the positive control, but there was no significant migration of either subset to the different secretomes (Friedman's 1 way ANOVA – black lines on graphs), all statistical results are shown on the respective graphs. Transwell migrations in 2 donors were performed on sorted CXCR3+ NK cells (blue bars), CXCR3- NK cells (yellow bars) and unsorted polyclonally activated NK cells (Green bars) to the secretomes (C), depleting CXCR3 expressing activated NK cells prevents migration to Latent monocyte infected secretomes.

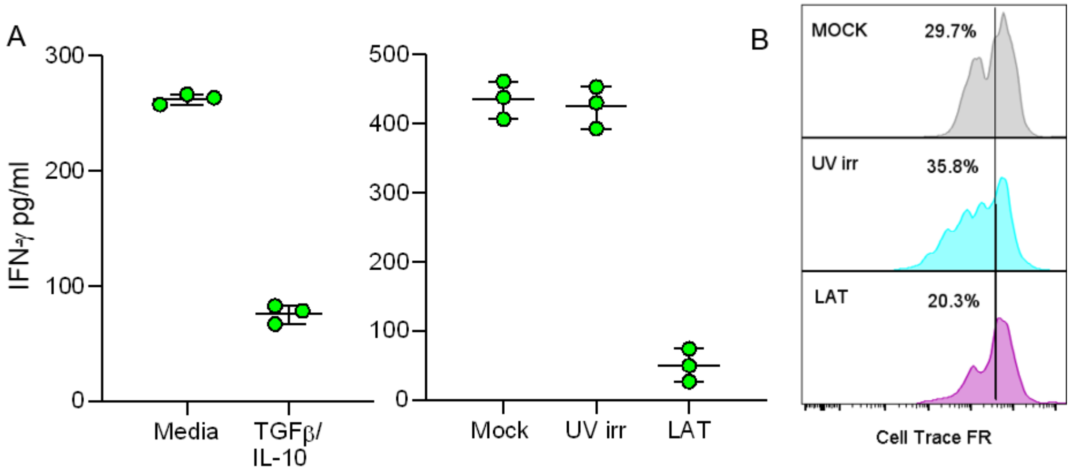

Figure S5 - Effects of Monocyte Latent infected secretomes on CMV specific CD4+ T cell functions

CD4+ T cells were resuspended in either X-VIVO Media (Media), media with added recombinant protein TGFβ & IL-10 (TGFβ/IL-10), Mock, UV irradiated or Latent infected secretome following 24 hours pre-treatment the CD4+ T cells were stimulated with a mixture of peptides from CMV gB protein for a further 24 hours and supernatants harvested. Production of IFNγ in the supernatants was measured by ELISA, representative results from one donor analyzed are shown (A). Histograms from a proliferation assay ran in parallel and harvested after 6 days stimulation are shown (B) there is suppression of proliferation in the latent secretome treated CD4+ T cells (Pink filled histogram).

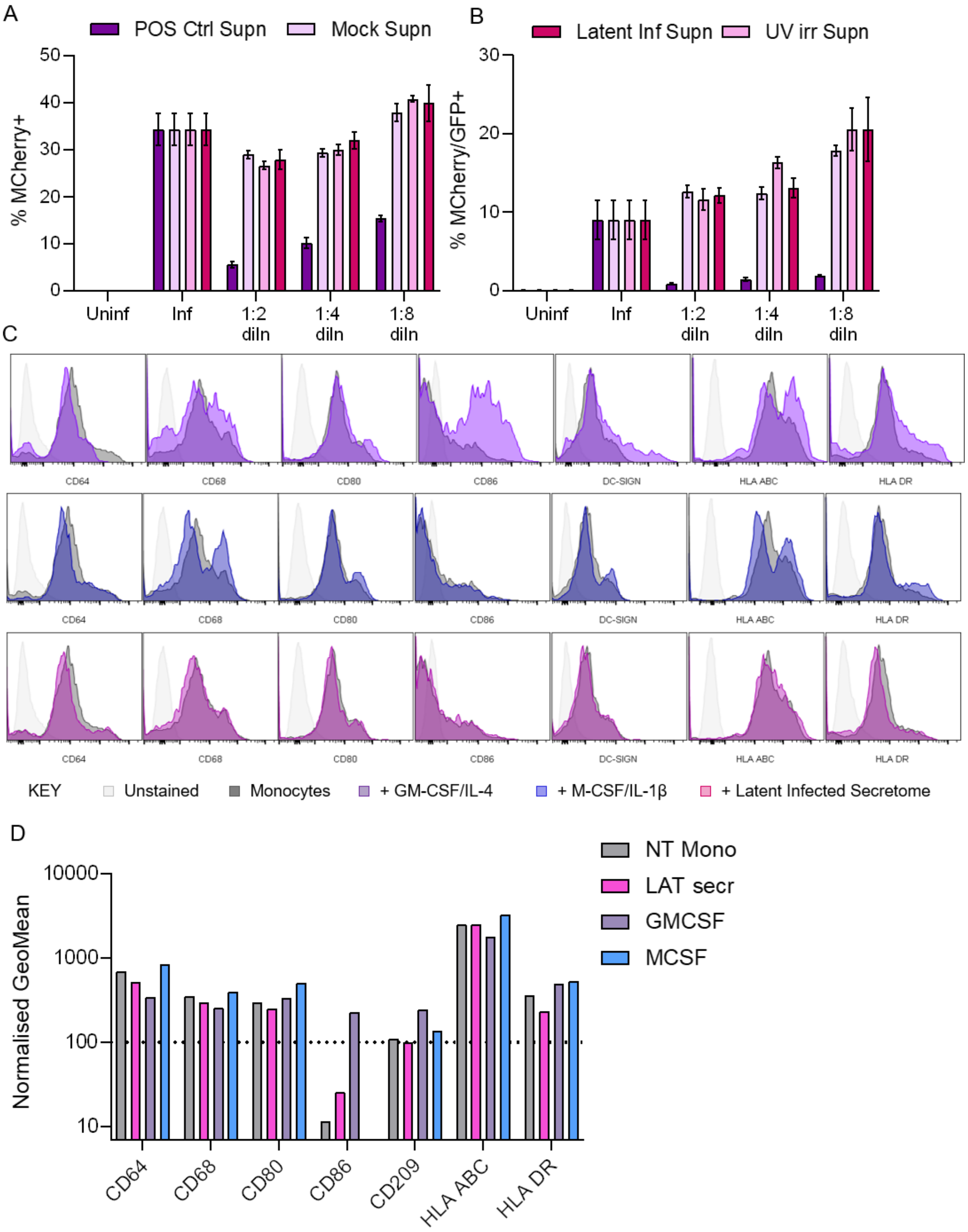

Figure S6- Effects of Monocyte Latent infected secretomes on lytic viral dissemination and bystander monocyte differentiation phenotype

HFFFs were infected with Merlin strain modified to express protein UL36-mCherry and protein UL32-GFP at a low MOI; after 24 hours a positive control supernatant, mock infected, UV irradiated and latent infected CD14+ supernatants were added at a range of dilutions and then incubated for 10 days. A bar charts summarizing the results for the percentage of HFFFs expressing UL36-mCherry (A) and UL32-GFP (B) are shown, whilst the positive control supernatant controlled the dissemination of virus none of the monocyte supernatants had any effect. Flow cytometry analysis of the expression of CD64, CD68, CD80, CD86, CD209 (DC-SIGN), MHC Class I (HLA ABC) and MHC Class II (HLA-DR) was performed for untreated monocytes (dark grey filled histogram), GM-CSF & IL-4 treated (purple filled histogram), M-CSF & IL-1β treated (blue filled histogram) or Latent infected monocyte secretome treated monocytes (pink filled histogram) is shown (C). The expression of each marker was normalized to isotype control-stained monocytes and the relative expression measured using the geometric mean (GeoMean) for monocytes (grey), Latent secretome treated (pink – LAT secr), and cytokine GMCSF (mauve) and MCSF (blue) treated monocytes are graphed (D). Treatment of monocytes with differentiation cytokine cocktails resulted in increased expression of MHC Class II, and other co-stimulatory molecules associated with myeloid differentiation e.g. increased expression of DC-SIGN in the GM-CSF treated monocytes; but the latent infected monocyte secretome did not alter the phenotype of the monocytes compared to untreated monocytes.

FIGURE S7

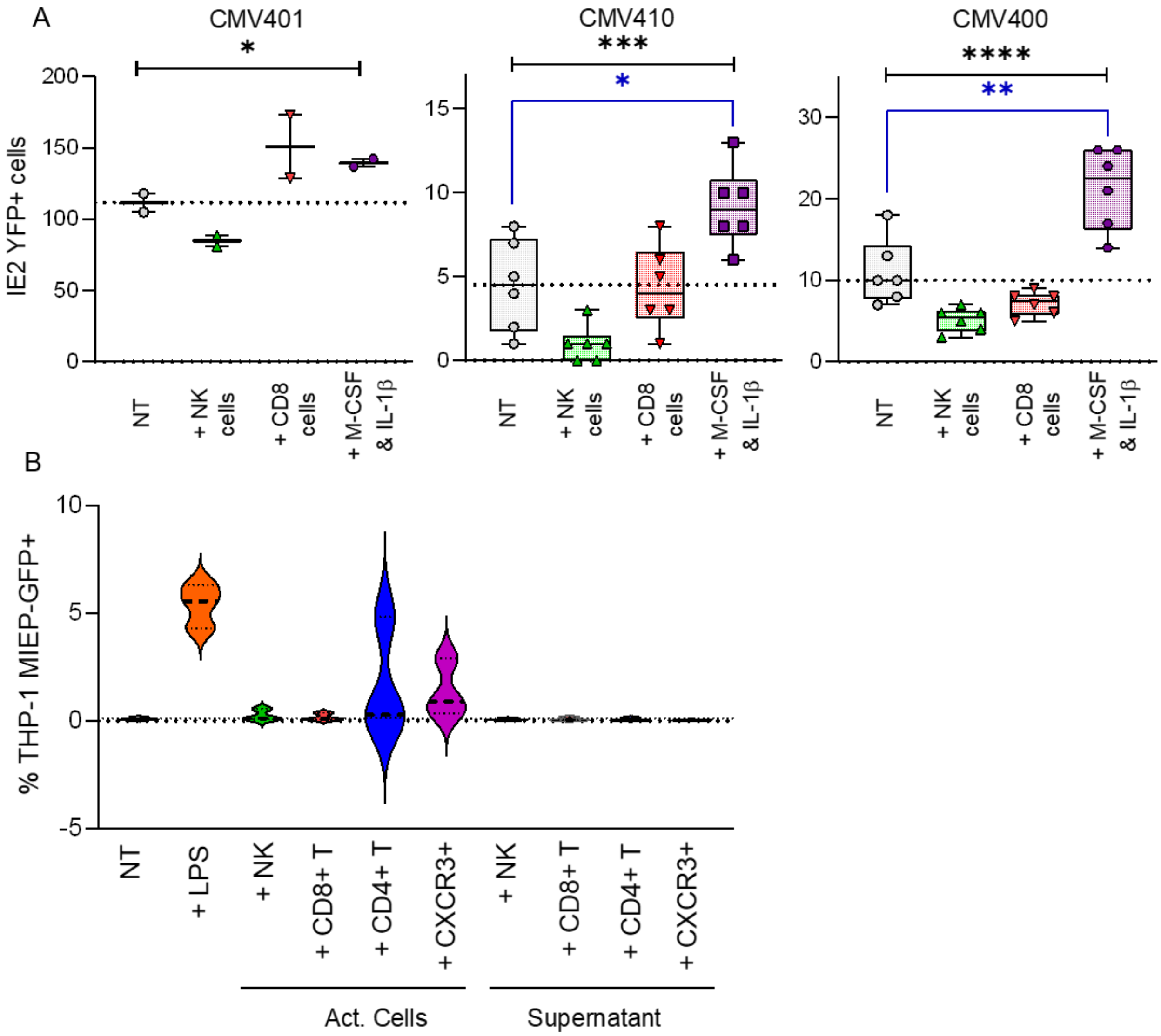

Figure S7 - Activated CD8+ T cells and NK cells and supernatants from activated lymphocyte subsets do not reactivate virus

CD14+ monocytes were infected with TB40/E IE2-YFP virus and co-cultured with activated NK cells, CD8+ T cells or treated with M-CSF & IL-1β cytokine cocktail in 3 separate donors, reactivating i.e. YFP positive cells were visualized by microscope and enumerated (A), only significant levels of YFP expressing cells were detected in cytokine treated monocytes (Kruskal Wallis 1-way ANOVA (black line CMV401 p=0.0317; CMV410 p=0.0003; CMV400 p<0.00010 and Dunn's post-test (blue lines CMV410 M-CSF p=0.0133; CMV400 M-CSF p=0.0034)).

THP-1 monocytes with an integrated MIEP driving GFP expression were co-cultured with supernatant from activated NK, CD8+ T, CD4+ T or CXCR3+ PBMC cells or the corresponding_activated cell

subsets for 7 days. GFP expression in the cell line was then analyzed by flowcytometry in comparison to untreated and LPS treated (positive control) cells, the results are displayed as a violin plot with mean of 3 replicates for each condition indicated (B).

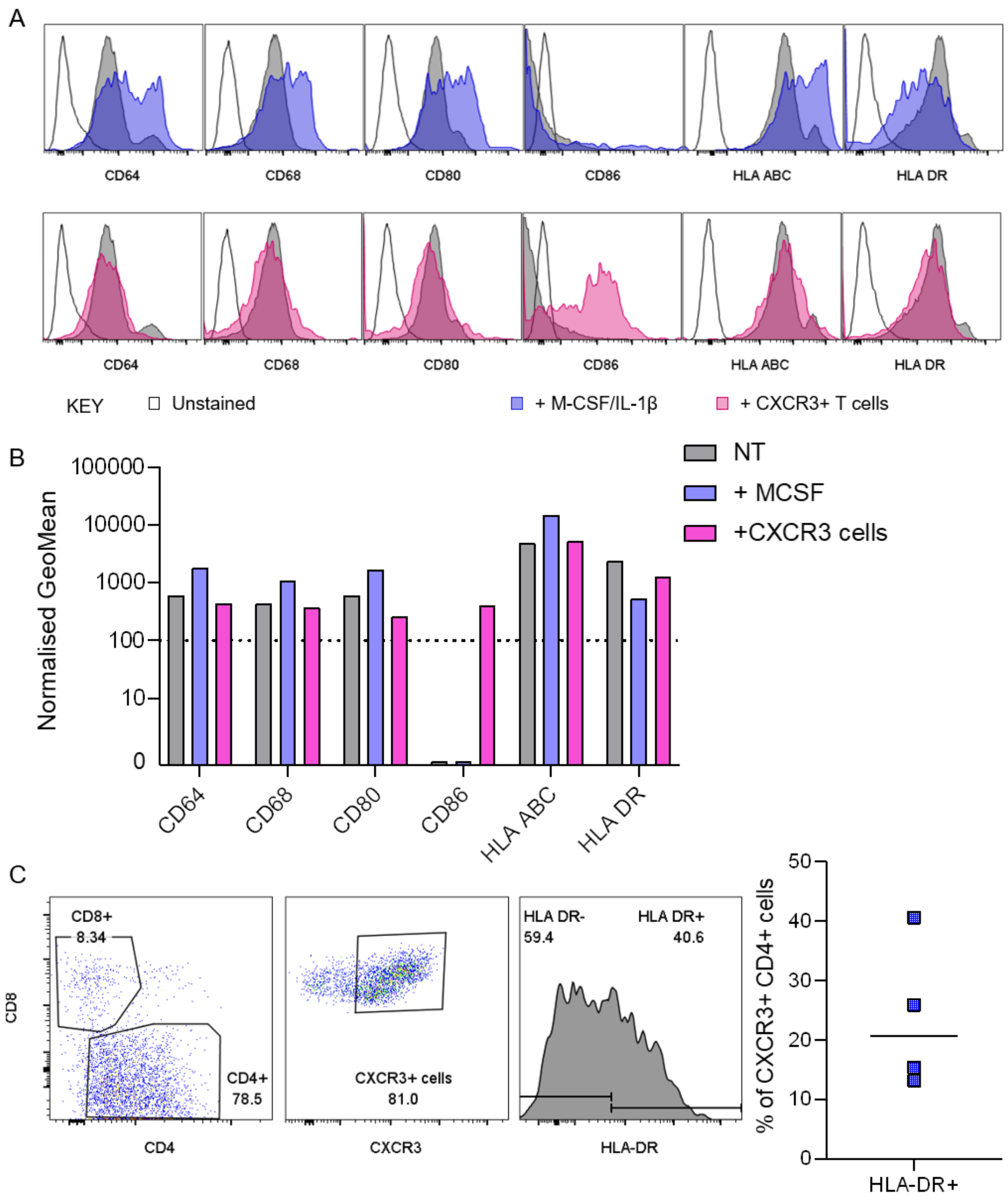

Figure S8 - Expression of myeloid differentiation markers are increased on monocytes co-cultured with CXCR3+ T cells & upregulation of HLA-DR expression on CXCR3+ CD4+ T cells.

Flow cytometry analysis of the expression of cell surface markers either increased or decreased during myeloid differentiation were analyzed. Histograms depicting the expression of CD64, CD68, CD80, CD86, MHC Class I (HLA ABC) and MHC Class II (HLA-DR) are shown for untreated monocytes (grey filled histogram) M-CSF & IL-1β treatment (blue filled histogram) and CXCR3+ T cell co-culture (pink filled histogram) (A). The expression of each marker was normalized to the isotype

control stained monocytes and the relative expression measured using the geometric mean (GeoMean) for monocytes (grey), MCSF treated monocytes (blue) and CXCR3+ co-cultured monocytes (Pink) are graphed (B).

Flow cytometry phenotype analysis of the co-expression of HLA-DR on CXCR3+ CD4+ T cells are shown, with representative staining from 1 donor (Left Hand side dot plots and histogram) and a summary graph showing the expression of HLA-DR on CXCR3+ CD4+ T cells in 4 different donors (right hand side) (C).

FIGURE S9

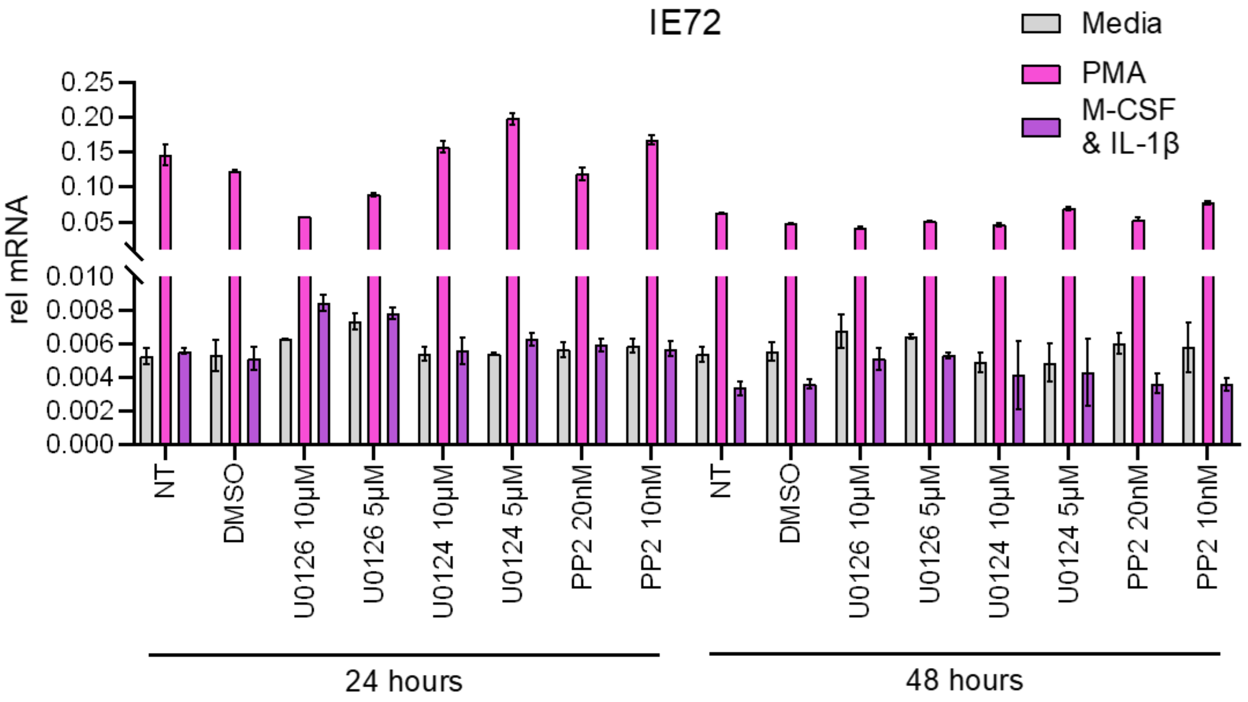

Figure S9 - Inhibition of ERK signaling pathways in monocytes to prevent reactivation

Inhibition of ERK signaling pathway was interrogated using U0126 a MEK inhibitor and PP2 a Src family kinase inhibitor. Latent monocytes were treated with either Media, PMA or M-CSF/IL-1β in the presence of DMSO, inhibitor U0126 or its inactive control U0124, or inhibitor PP2.

3 References

1. Poole E, Avdic S, Hodkinson J, Jackson SE, Wills M, Slobedman B, et al. Latency-associated viral interleukin-10 (IL-10) encoded by human cytomegalovirus modulates cellular IL-10 and CCL8 Secretion during latent infection through changes in the cellular microRNA hsa-miR-92a. Journal of virology. 2014;88(24):13947-55.
2. Goodrum F, Reeves M, Sinclair J, High K, Shenk T. Human cytomegalovirus sequences expressed in latently infected individuals promote a latent infection in vitro. Blood. 2007;110(3):937-45.
3. Pfaffl MW. A new mathematical model for relative quantification in real-time RT-PCR. Nucleic Acids Res. 2001;29(9):e45.
4. Human Cytomegaloviruses: Methods and Protocols. 2nd ed: Humana Press; 2021. XI, 469 p.
5. Poole E, Groves I, Jackson S, Wills M, Sinclair J. Using Primary Human Cells to Analyze Human Cytomegalovirus Biology. Methods Mol Biol. 2021;2244:51-81.
6. Dupont L, Du L, Poulter M, Choi S, McIntosh M, Reeves MB. Src family kinase activity drives cytomegalovirus reactivation by recruiting MOZ histone acetyltransferase activity to the viral promoter. The Journal of biological chemistry. 2019;294(35):12901-10.
